# Rifaximin Modulates the Gut Microbiota to Prevent Hepatic Encephalopathy in Liver Cirrhosis Without Impacting the Resistome

**DOI:** 10.3389/fcimb.2021.761192

**Published:** 2022-01-18

**Authors:** Xiao Yu, Ye Jin, Wangxiao Zhou, Tingting Xiao, Zhongwen Wu, Junwei Su, Hainv Gao, Ping Shen, Beiwen Zheng, Qixia Luo, Lanjuan Li, Yonghong Xiao

**Affiliations:** ^1^ State Key Laboratory for Diagnosis and Treatment of Infectious Diseases, National Clinical Research Center for Infectious Diseases, First Affiliated Hospital, School of Medicine, Zhejiang University, Hangzhou, China; ^2^ Department of Respiratory and Critical Care Medicine, First Hospital of Shanxi Medical University, Taiyuan, China; ^3^ Department of Infectious Disease, ShuLan (Hangzhou) Hospital Affiliated to Zhejiang Shuren University Shulan International Medical College, Hangzhou, China

**Keywords:** liver cirrhosis, gut microbiota, metagenomics, resistome, rifaximin

## Abstract

The gut microbiota has an important role in the pathogenesis of hepatic encephalopathy(HE). Rifaximin, an intestinal non-absorbable antibacterial agent, is effective in the treatment of HE. However, whether long-term prophylactic use induces antibacterial resistance and its mechanism for treating HE remains unclear. This prospective study assessed the impact of 12 weeks rifaximin administration on the gut microbiota and resistome in cirrhotic patients. Fecal sampling was conducted 1 day before the first rifaximin administration and at Weeks 1, 2, 4, 6, 8, 10, 12 of the study. Thirty cirrhotic patients who were in remission from recurrent HE was enrolled to receive rifaximin (400mg TID for 12 weeks). Rifaximin improved hyperammonemia and cognitive function in the 21 patients who completed rifaximin treatment. The dynamic observations showed the gut microbiota diversity, composition and the number of resistance genes, plasmids, insertion sequences did not change significantly during the period(P>0.05). Metabolic pathways such as aromatic amino acids, tryptophan synthesis, urea cycle, and LPS synthesis reduced. No new antimicrobial resistance genes was emergenced. However, the number of aminoglycosides, rifamycin and phenolic resistance genes increased, whereas tetracycline, fosfomycin and cephamycin decreased (P<0.05). Changes in the abundance of *E. coli, K. pneumoniae*, and *B. longum* strains correlated with changes of resistance genes. Prophylactic use of rifaximin for 12 weeks improved hyperammonemia and neurophysiological function, maintained gut microbiota diversity, composition and did not change the overall resistome. Rifaximin altered expression of HE-related metabolic pathways. All of these effects could play a key role in preventing HE. **Clinical Trial Registration**: ChiCTR1900022234 (registered at the Chinese Clinical Trial Registry).

## Introduction

Chronic liver disease is common, and hepatic encephalopathy(HE) is a common serious complication with a high mortality rate in patients with end-stage cirrhosis. The main drugs currently used for prevention and treatment of HE are L-ornithine-L-aspartate, lactulose, lactitol, and rifaximin. Rifaximin(as the only available crystalline α form rifaximin in clinical, rifaximin refers to rifaximin-α in this article) is a rifamycin derivative with low absorption that may exert pharmacological actions through its effect on the gut microbiota (Kogawa and Salgado, 2018). In 2010, the US FDA approved rifaximin for prevention and treatment of HE; indeed, EASL-AASLD guidelines (2014 version) for the treatment of HE recommends this drug as an effective add-on treatment for patients with overt HE (i.e., those who develop the disease while taking lactulose) and for preventing recurrence of HE. In addition, the Consensus Opinion on the Diagnosis and Treatment of HE in China (2013) recommends rifaximin as a widely available drug for preventing the recurrence of HE (FDA Approves New Use of Xifaxan for Patients With Liver Disease, 2010); Chinese Society of Hepatology Chinese Medical Association, 2013; American Association for the Study of Liver Diseases and European Association for the Study of the Liver, 2014). The mechanism by which rifaximin prevents and treats of HE is still not fully understood.

The human gut is rich in microorganisms, which play an important role in maintaining homeostasis in the body (Ley et al., 2006; Brenchley Jason and Douek Daniel, 2012; Acharya et al., 2017). Antibiotics such as vancomycin, imipenem, and ciprofloxacin have been reported to extremely reduce the diversity and increase antibiotic resistance in the gut microbiota (Morgun et al., 2015; Gibson Molly et al., 2015; Choo et al., 2017). Rifaximin is an antimicrobial drug that is not absorbed from the gut; therefore, it accumulates at high concentrations in the gut after oral administration. The effect of rifaximin on the gut microbiota and resistome after long-term prophylactic and therapeutic use for HE is unclear. Studies based on 16s rRNA gene amplicon sequencing report that rifaximin does not affect gut microbiome diversity, and that it has very little effect on bacterial abundance in the microbiome (Bajaj et al., 2013). Therefore, it is hypothesized that changes in bacterial metabolic function, rather than changes in bacterial populations, underlie the primary mode of action of rifaximin for treating HE. In addition, serum metabolomics show an increase in serum linoleic, linolenic, and arachidonic unsaturated fatty acid content, and a decrease in lipopolysaccharide content, after rifaximin treatment, suggesting that rifaximin changes metabolic function in the gut microbiota (Bajaj et al., 2013; Kaji et al., 2017). However, 16s rRNA amplicon sequencing has limited resolution, and so gut microbiota are generally annotated to the genus level (rather than the species level); by contrast, the application of metagenome sequencing allows independent analysis of changes in gut microbiota populations and functions (Lepage et al., 2013; Bajaj and Khoruts, 2020).

Antimicrobial resistance has become a serious public health crisis; the emergence of multi-drug resistance and pan-drug resistance bacterial is common in clinical practice, resulting in bacterial infections that are often untreatable and pose a serious threat to life. Indeed, antimicrobial resistance has attracted great attention from the World Health Organization and national governments (Metcalfe et al., 2016; Xiao, 2018; Zhou et al., 2018; Wu et al., 2019). The gut contains a wide variety of bacteria and can be considered an antimicrobial resistance gene pool. The effects of various antimicrobial drugs on gut bacterial resistance have been reported widely, with the use of meropenem, ticarcillin/clavulanate, cefotaxime, gentamicin, and vancomycin increasing the number of their corresponding resistance genes in the gut microbiota (Gasparrini et al., 2016). The effect of long-term prophylactic use of rifaximin on gut microbiota resistance in patients with cirrhosis also deserves an in-depth study to avoid worsening of resistance.

The purpose of this study was to investigate dynamic changes in the gut microbiota of cirrhotic patients during 12 weeks rifaximin administration, and to study possible therapeutic mechanisms of rifaximin-α, as well as to understand the effect of rifaximin on gut antimicrobial resistances genes and to make recommendations for the rational clinical use of rifaximin in cirrhotic patients.

## Patients and Methods

### Study Design and Patient Enrollment

This prospective study was conducted in accordance with the Declaration of Helsinki and approved by the ethics committee of the First Affiliated Hospital, College of Medicine, Zhejiang University, China (No:RN.2017.665). Eligible cirrhotic patients were enrolled to receive rifaximin(400mg TID for 12 weeks). All patients were treated routinely according to the guidelines for the diagnosis and treatment of cirrhosis (Chinese Society of Hepatology Chinese Medical Association, 2013). Additional protocols and inclusion/exclusion criteria are described in the supplement materials.

### Specimen Collection, Genome Extraction, and Metagenomics Sequencing

Stool specimens were collected one day before drug administration and at week 1, 2, 4, 6, 8, 10 and 12 weeks of the study, ensuring that each specimen weighed more than 1 g. The genomes of bacteria present in stool samples were extracted using a commercially available kit (Qiagen), according to the manufacturer’s instructions. The DNA concentration of the extracted genome was measured and sequenced on an Illumina Novseq 6000 instrument, with 10G of sequencing data per sample.

### Taxonomic, Functional Profiling and Correlation Analysis of Gut Microbiota and Resistance Genes

Taxonomic profiling of the stool samples was performed using MetaPhlAn2 v2.5.0 with default settings. Abundance of metabolic pathways was performed with HUMAnN2v0.9.4. The antimicrobial resistance genes analysis was performed using the bwt function in the rgi software package. The correlation between gut microbiota and resistance genes was based on co-occurrence analysis. The “igraph” and “Hmisc” packages in R language were used to calculate the correlation matrix between resistant genes and strains by WGCNA (weighted gene co-expression network analysis). Correlation matrix R values and correlation matrix p values between resistance genes and strains were calculated, and p values were adjusted using the Benjamini-Hochberg method correction. The above matrix was graphically displayed using Gephi software.

### Statistical Methods

Measurement data were expressed as the mean ± standard deviation $\left(\bar{x}\pm \text{s}\right)$ . Data that were normally distributed were tested using either a Student’s t-test (including independent and paired samples), Fisher’s exact test, or an ANOVA test. A non-parametric Wilcoxon test was used for non-normally distributed data. A chi-square test was used for the count data. The ANOSIM (analysis of similarities) non-parametric test was used to analyze differences in principal coordinate analysis. Data were processed using R statistical software. A P-value < 0.05 was considered statistically significant.

### Data Availability Statement

Metagenomic sequencing data for all samples have been deposited in the NCBI Sequence Read Archive database under accession numbers PRJNA689208 (https://www.ncbi.nlm.nih.gov/bioproject/PRJNA689208).

## Results

### Characteristics of the Study Population

**Figure 1:**
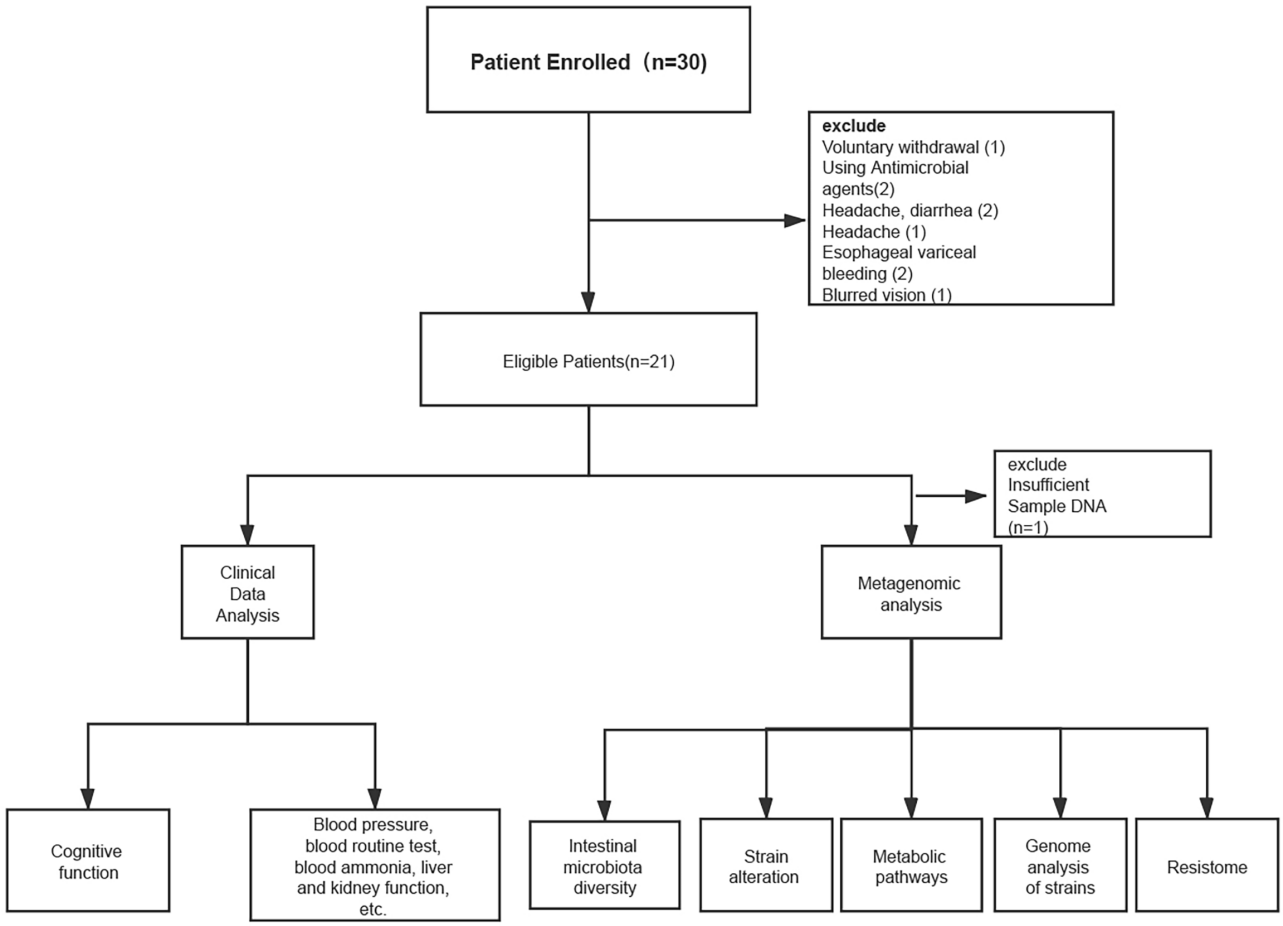
Patient enrollment and research flow chart.

Thirty cirrhotic patients were enrolled in the study from March, 2018 to May, 2019, all of whom belonged to CTP(Child-Turcotte-Pugh) class A; 21 patients completed the study protocol (**Figure 1**). Stool samples from 20 patients underwent metagenomic analysis (one patient had insufficient DNA in their stool sample for metagenomic sequencing) (**Figure 1**).

Basic patient information and patient medication use during the study period were described in the supplemental material (**Supplementary Table 1**). No significant changes in blood pressure, heart rate, alpha-fetoprotein levels, hepatitis B markers, CA-199 levels, CA-125 levels, liver and kidney function, or routine blood and urine test results were observed during the study (**Supplementary Table 2**). Blood ammonia concentrations fell gradually in patients without HE during the study period (from 54.1 ± 14.7 μmol/L to 25.6 ± 9.7 μmol/L; P < 0.05). There was a statistically significant improvement in the time taken by patients to complete NCT-A(number connection test) scores (from 121.4 ± 20.3 s to 112.3 ± 18.4; P < 0.05) and DST(digit symbol test) scores (8.3 ± 2.1 to 10 ± 3.1; P < 0.05) after rifaximin administration (**Table 1**).

**Table 1:** Results of ammonia measurement and cognitive test scores in patients during rifaximin-α administration.

Group	Content	Start day	Week 1	Week 4	Week 8	Week 10	Week 12
No HE (n=16)	Ammonia (μmol/L)	54.1±14.7	42.1±8.3*	47.5±5.6*	35.3±8.6*	31.2±7.3*	25.6±9.7*
	NCT-A (s)	121.4±20.3	118.4±10.5*	111.4±17.3*	105.4±20.3*	108.1±30.3*	112.3±18.4*
	DST	8.3±2.1	9.2±1.4*	9.1±3.1*	9.8±2.4*	9.3±2.1*	10.1+3.1*
HE(n=5)	Ammonia (μmol/L)	52.8±10.3	47.6±9.8*	52.8±8.9	68.2±16.7*	69±19.7*	44±10.1*
	NCT-A (s)	123±6.7	128.6±10.2	134±15.1*	152.2±24.7*	159.8±19.4*	133.8±15.7*
	DST	8.2±1.3	8.2±3.1	7.8±4.7	6.6±6.9*	6±8.7*	6.8±2.8*

* Statistically significant difference compared with the starting day (p<0.05).

Four patients experienced transient diarrhea during the dosing period, which improved without treatment. Nine patients underwent transjugular intrahepatic portosystemic shunt (TIPS), of which four developed HE during the drug administration regimen. One patient in the non-TIPS group (comprising 12 patients) developed HE during drug administration.

### Gut Microbiota Composition in Patients With Cirrhosis and the Impact of Rifaximin

**Figure 2:**
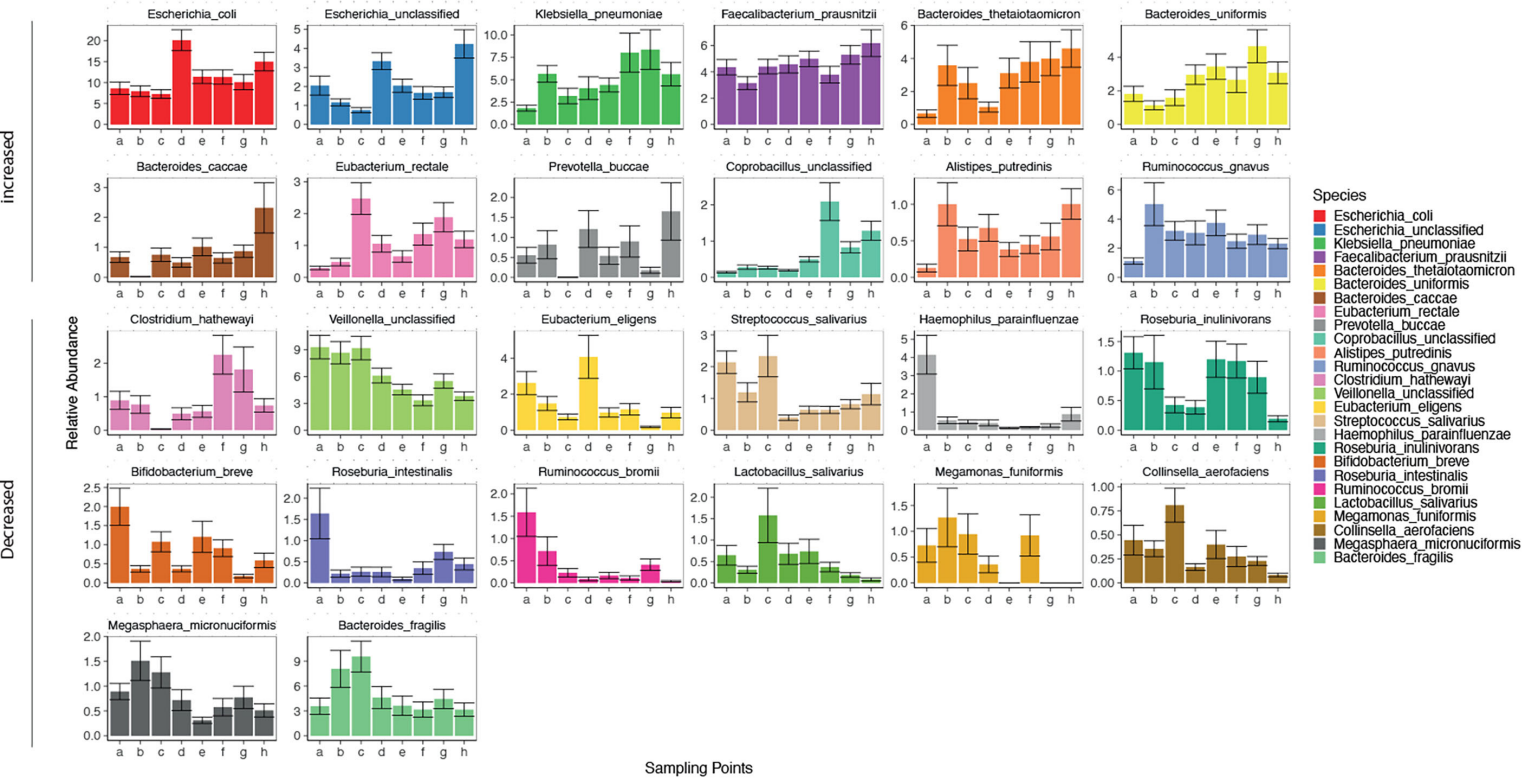
Changes in gut microbiota (at a species level) in patients over time. Horizontal coordinates denote sampling time points; a–h represent Day 1, Week 1, Week 2, Week 4, Week 6, Week 8, Week 10, and Week 12, respectively. Vertical coordinates denote the relative abundance of the species (%).

**Figure 3:**
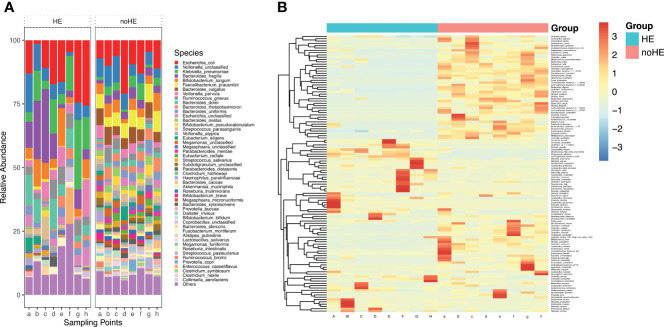
Comparison of, and changes in, the gut microbiota (at the species level) in patients with and without HE. **(A)** Composition of gut microbiota in patients with and without HE. **(B)** Changes in the abundance of 119 species of bacteria in patients with (left side) and without (right side) HE after rifaximin administration. Horizontal coordinates denote sampling time points; a–h represent Day 1, Week 1, Week 2, Week 4, Week 6, Week 8, Week 10, and Week 12, respectively.

The gut microbiota of the patients included in this study was comparable to the results of previous studies (Qin et al., 2014). As 11 phyla(184 genera, 506 species) were identified in the gut microbiota of the patients in this study. The dominant phylum(genera, species) present in the gut microbiota differed in each patient (**Supplementary Figure 1A**, and **Figures 2**, **3**). There was a decreasing trend in the abundance of *Firmicutes* and an increase in the abundance of *Proteobacteria* during rifaximin administration (**Supplementary Figure 1B**). Rifaximin caused a decreased trend in the abundance of *Veillonella, Haemophilus, Streptococcus, Parabacteroides, Megamonas, Roseburia, Alistipes, Ruminococcus*, and *Lactobacillus*. There was an increasing trend in the abundance of *Blautia*, *Fusobacterium*, *Prevotella*, and *Klebsiella* caused by rifaximin treatment at the genus level (**Supplementary Figure 2B**). At the species level, there was a trend toward an increase in abundance of *Escherichia. coli*, *unclassified Escherichia, Klebsiella.pneumoniae, Faecalibacterium prausnitzii, Bacteroides thetaiotaomicron*, and *Bacteroides. uniformis*, and a trend toward a decrease in the abundance of *Bifidobacterium breve*, *Roseburia intestinalis, Ruminococcus bromii, Megamonas funiformis*, and *Collinsella aerofaciens* (p<0.05) during the dosing period (**Figure 2**). The abundance of the remaining species fluctuated during the treatment period.

### Comparison of Gut Microbiota in Patients With and Without HE

The *Firmicutes, Candidatus Saccharibacteria*, and the *Verrucomicrobia* phyla were significantly lower in patients with HE (P<0.05) than in patients without HE, whereas *Bacteroidetes* and *Proteobacteria* were significantly higher in patients with HE (P<0.05) than in patients without HE (**Supplementary Figure 4**). The diversity of the gut microbiota was significantly lower in HE patients than in non-HE patients (P <0.05). There were 119 species differences between the two groups of patients (P<0.05), including 43 species that were present in higher abundance in HE patients and 76 strains that were higher in patients who did not develop HE (**Supplementary Table 3**). These 43 species mainly included *K. pneumoniae, K. oxytoca, unclassified Klebsiella, E. faecalis, E. cloacae, E. faecalis, C. freundii, R. aquatilis*, and *S. mitis oralis* (**Figure 3B**). Of the 43 species that were high in HE patients, 23 increased further during the study period in HE patients, but decreased in patients without HE. The 76 species that were present in high abundance in non-HE patients included *L. vaginalis*, *F. prausnitzii, R. obeum, A. muciniphila, B.pseudocatenulatum*, and *B. breve*, and the abundance of these species remained stable during the dosing period (**Figure 3B**).

### Effect of Rifaximin on the Diversity of the Gut Microbiota

**Figure 4:**
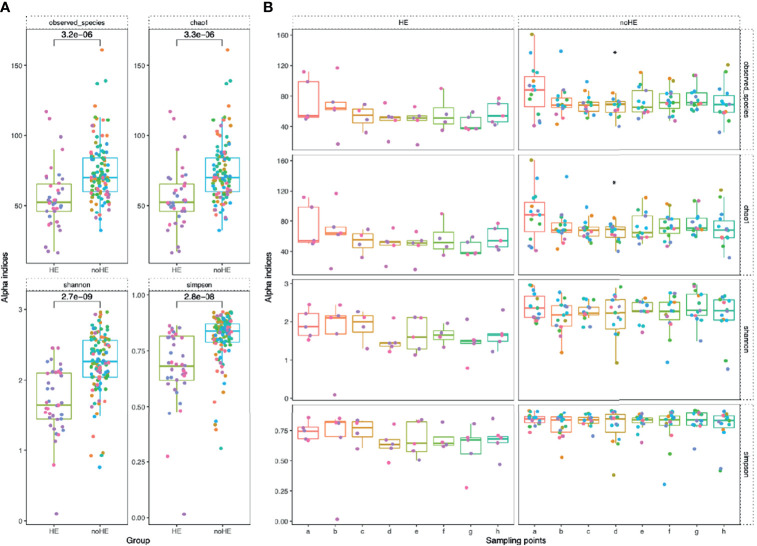
Comparison of microbiota diversity (at the species level) in patients with and without HE. Each point in the figure represents a patient: individual colors represent an individual patient. **(A)** Comparison of the overall gut microbiota diversity in patients with and without HE. The diversity of the microbiota was significantly lower in patients with HE than in patients without HE. The numbers in the figure show the statistical P-values between the two groups. **(B)** Changes in gut microbiota diversity between patients with and without HE during drug administration. Horizontal coordinates denote sampling time points, with a–h representing Day 1, Week 1, Week 2, Week 4, Week 6, Week 8, Week 10, and Week 12, respectively. Vertical coordinates represent the four indicators of bacterial diversity.

The diversity indices of the gut microbiota were calculated at the species level. Rifaximin treatment reduced both the number of species and the chao1 index in the gut microbiota only at Week 4; the number of observed species was not changed at other time points. The Shannon and Simpson indices did not change significantly during drug administration (maintained at 2.3 ± 1.5 and 0.8 ± 0.07, respectively; **Supplementary Figure 5A**). Based on principal coordinate analysis, the composition of the patient gut microbiota did not change significantly [as measured by the ANOSIM test, which did not show significant differences; R=-0.029, P>0.995 (**Supplementary Figure 5B**)]. The diversity of the gut microbiota was significantly lower in patients who experienced episodes of HE than in those who did not (P<0.05) (**Figure 4A**). The diversity of the gut microbiota (number of observed species, chao1 index, Shannon index, and Simpson index) remained stable during rifaximin administration in patients who did not develop HE (P>0.05), whereas there was a non-significant trend toward decreased diversity (mean 76.6 ± 5.1 to 53.3 ± 10.1) in patients who developed HE (**Figure 4B**). In addition, there was a significant decrease in the diversity of the microbiota at the time of HE occurrence (the Shannon index decreased by 0.2 to 0.8), followed by a gradual recovery (**Supplementary Figure 6**).

### Altered Metabolic Pathways in the Gut Microbiome Associated With HE

**Figure 5:**
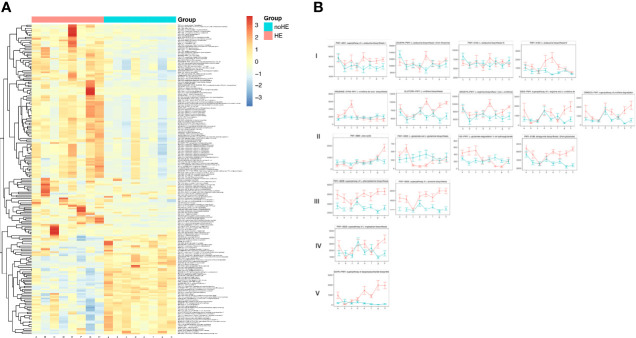
Changes in metabolic pathways of the gut microbiome associated with HE. **(A)** Changes in metabolic pathways of gut microbiota in patients with and without HE. **(B)** Changes in HE-related metabolic pathways in the gut microbiota of patients with and without HE I–V occurred in five main classes of pathway: increased synthesis of branched-chain amino acids (type I); pathways related to ammonia metabolism (type II); synthesis of aromatic amino acids (type III); synthesis of tryptophan (type IV); and synthesis of LPS (type V). Horizontal coordinates denote sampling times, with a–h corresponding to Day1, Week 1, Week 2, Week 4, Week 6, Week 8, Week 10, and Week 12 of the study, respectively. Vertical coordinates denote the abundance of pathways, expressed as RPK (number of reads per Kb base sequence). Red lines represent patients with HE and green lines represent patients.

Among the 535 metabolic pathways identified, there were 206 differences (P<0.05) between patients who suffered HE and those who did not, of which 67 pathways were decreased in patients with HE and 139 pathways were increased (**Supplementary Table 4** and **Figure 5A**). These differential metabolic pathways were mainly involved in synthesis of phosphopeptidic acid, peptidoglycans, amino acids, and fatty acids; in degradation of alcohols; in degradation of rockulose and rhamnose; and in synthesis and degradation of coenzymes. Thirty-nine metabolic pathways related to HE were selected for analysis; the results showed that 17 pathways related to HE patients increased during rifaximin administration. These pathways could be classified as five main types: synthesis of branched-chain amino acids (type I, n=4); pathways related to ammonia metabolism (type II, n=9); synthesis of aromatic amino acids (type III, n =2); increased synthesis of tryptophan (type IV, n=1); and synthesis of LPS (type V, n=1) (**Figure 5B**). These selected pathways remained stable in non-HE patients.

### Genomic Variation of Gut Strains Under the Influence of Rifaximin

A total of 35 species with genome sequencing coverage >20% and sequencing depth >15X showed genomic variation. COG annotation of SNV-mutated genes revealed that about 10.1% of SNV genes were related to gene transcription and 8.3% were related to bacterial replication and repair functions, whereas 6.1% of mutations occurred in genes related to glycans, 6.0% occurred in genes related to lipid transport, and 4.1% occurred in genes related to energy metabolism (**Supplementary Figure 7**); these mutations may lead to changes in expression of metabolic pathways. After administration of rifaximin, the species found to have substitutions based on SNV after WSS calculations included *E. coli, B. longum, C. aerofaciens, V. parvula, P. prausnitzii, L. salivarius, L. fermentum*, and *B. uniformis*. Some of these strains will be stable in patients after substitution, including *B. capillosus, A. shahii*, and *R. gnavus*, whereas some will be replaced by the original strains after substitution, including *L. fermentum, C. aerofaciens*, and *V. parvula* (**Supplementary Figure 8**). Based on the grid growth rate index calculated by SNV, *E. coli, B. longum, C. aerofaciens, F. prausnitzii, R. gnavus, A. shahii*, and *B. uniformis* showed increased growth rates after rifaximin treatment. The growth rate of *V. parvula, C. bolteae, C. nexile, L. fermentum*, and *L. salivarius* was reduced after rifaximin treatment.

### Changes in the Gut Resistome After Rifaximin Treatment

**Figure 6:**
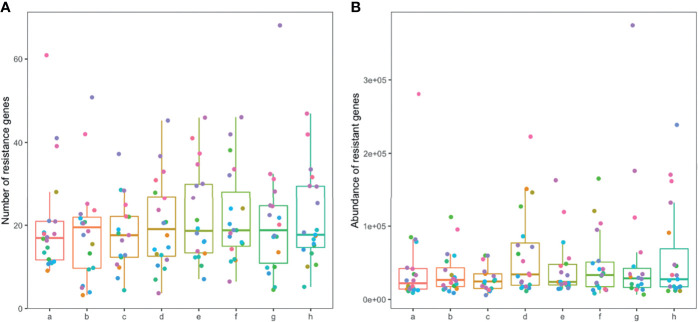
Number and abundance of antibiotic resistance genes in the gut microbiome of patients. Horizontal coordinates denote sampling time points: a–h represent Day 1, Week 1, Week 2, Week 4, Week 6, Week 8, Week 10, and Week 12, respectively. Vertical coordinates denote the number **(A)** or abundance **(B)** of resistance genes.

**Figure 7:**
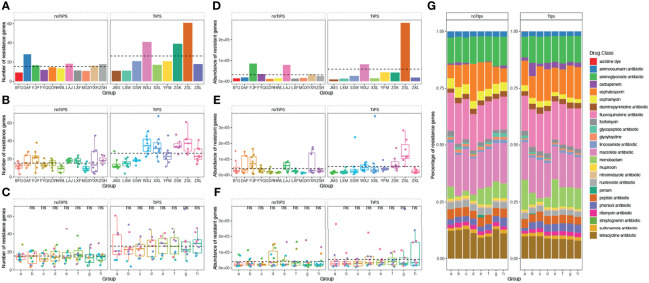
Number, abundance, and composition of drug resistance genes in the gut microbiome of TIPS and non-TIPS patients. **(A)** Comparison of the number of gut resistance genes per patient in the TIPS group and non-TIPS group before rifaximin administration. **(B)** Comparison of the number of gut resistance genes in the TIPS group and the non-TIPS group during rifaximin administration (seven samples per patient). **(C)** The change in the number of gut resistance genes in the TIPs group and the non-TIPS group during rifaximin administration. **(D)** Change in the number of gut resistance genes in the TIPs group and the non-TIPS group before rifaximin administration. **(E)** Comparison of the abundance of gut resistance genes in the TIPS group and the non-TIPS group during rifaximin administration (seven samples per patient). **(F)** The change in the abundance of gut resistance genes in the TIPs group and the non-TIPS group during rifaximin administration. **(G)** Composition of drug resistance genes in the gut microbiome of TIPS and non-TIPS patients. The dashed line in the graphs represents the mean. ns denotes no statistical difference compared with samples collected before drug administration.

A total of 1845 drug resistance genes were identified in the gut microbiome of patients; the number of drug resistance genes carried by each patient ranged from 23 to 343. The number and abundance of drug-resistant genes in the gut microbiome of patients remained stable during the drug administration period, and there was no statistical difference compared with the pre-drug period (**Figure 6**). The number of resistance genes carried by the gut microbiota of patients in the TIPS group (22.3 ± 5.7) was higher than that in the non-TIPS group (18.1 ± 4.5) (P<0.05), but there was no significant difference in the abundance of resistance genes between the two groups (P>0.05) (**Figures 7A, B, D, E**). The number and abundance of resistant genes did not change in either the TIPS or non-TIPS group after rifaximin treatment was initiated, and remained stable through the study period (**Figures 7C, F**). The composition of gut resistance genes was similar in the TIPS and non-TIPS groups, and it remained stable during rifaximin administration (**Figure 7G**).

### Changes in the Types of Antimicrobial Resistance Genes in the Gut of Cirrhosis Patients

Resistance genes are characterized according to the antimicrobial agent first found to mediate resistance. The stool samples of the patients in this study contained 24 types of antimicrobial resistance genes. Rifamycin-related resistance genes accounted for about 3.6%. Four types of change in resistance genes were identified during the dosing period. This first is a gradual increase, which was observed for genes conferring resistance to aminoglycosides, phenicol antibiotics, and rifamycin (P<0.05). The second change is a decrease and then an increase, which was observed for genes conferring resistance to lincosamides and glycopeptides. The third change is an increase and then a decrease, as seen for genes conferring resistance to cephalosporins and mupirocin. The fourth change is a gradual decrease, as observed for genes conferring resistance to tetracyclines and cephalosporins. (P<0.05) (**Supplementary Figure 9**). Moreover, TIPS patients carried significantly more resistance genes than non-TIPS patients, and the increase in the number of resistance genes, such as genes conferring resistance to aminoglycosides, rifamycins, and phenolics, occurred in the gut microbiota of TIPS patients during the study (P<0.05), while the numbers of these three types of resistance gene remained stable in non-TIPS patients (**Supplementary Figure 10**).

Clinically important carbapenemase genes such as *bla_NDM_, bla_KPC_, bla_VIM_*, and *bla_IMP_*, as well as the vanA and vanB vancomycin resistance genes, were not detected in cirrhotic patients in this study; however, the vanC, vanD, vanRG, and vanRC resistance genes were detected.

### Numbers and Types of Mobile Elements in the Gut Microbiome of Patients With Cirrhosis

A total of 95 plasmid types were carried in the gut microbiota of patients with cirrhosis, with the most abundant being IncFIB plasmids (accounting for 11.37% of all plasmids). Each patient carried 11–20 plasmids, with an average of 10.1 ± 3.9 plasmid types per patient, and carriage remained stable throughout the study period (**Supplementary Figure 11**). A total of 781 diverse IS were carried in the gut microbiota of cirrhotic patients, with no clear dominant type. Each patient carried 18–89 IS, with an average of approximately 1219.7 ± 301.4 IS sequences, and carriage remained stable and similar to the trend in plasmid carriage observed during rifaximin administration (**Supplementary Figure 12**).

### Correlation Between Gut Microbiota and Resistance Genes in Patients With Liver Cirrhosis

**Figure 8:**
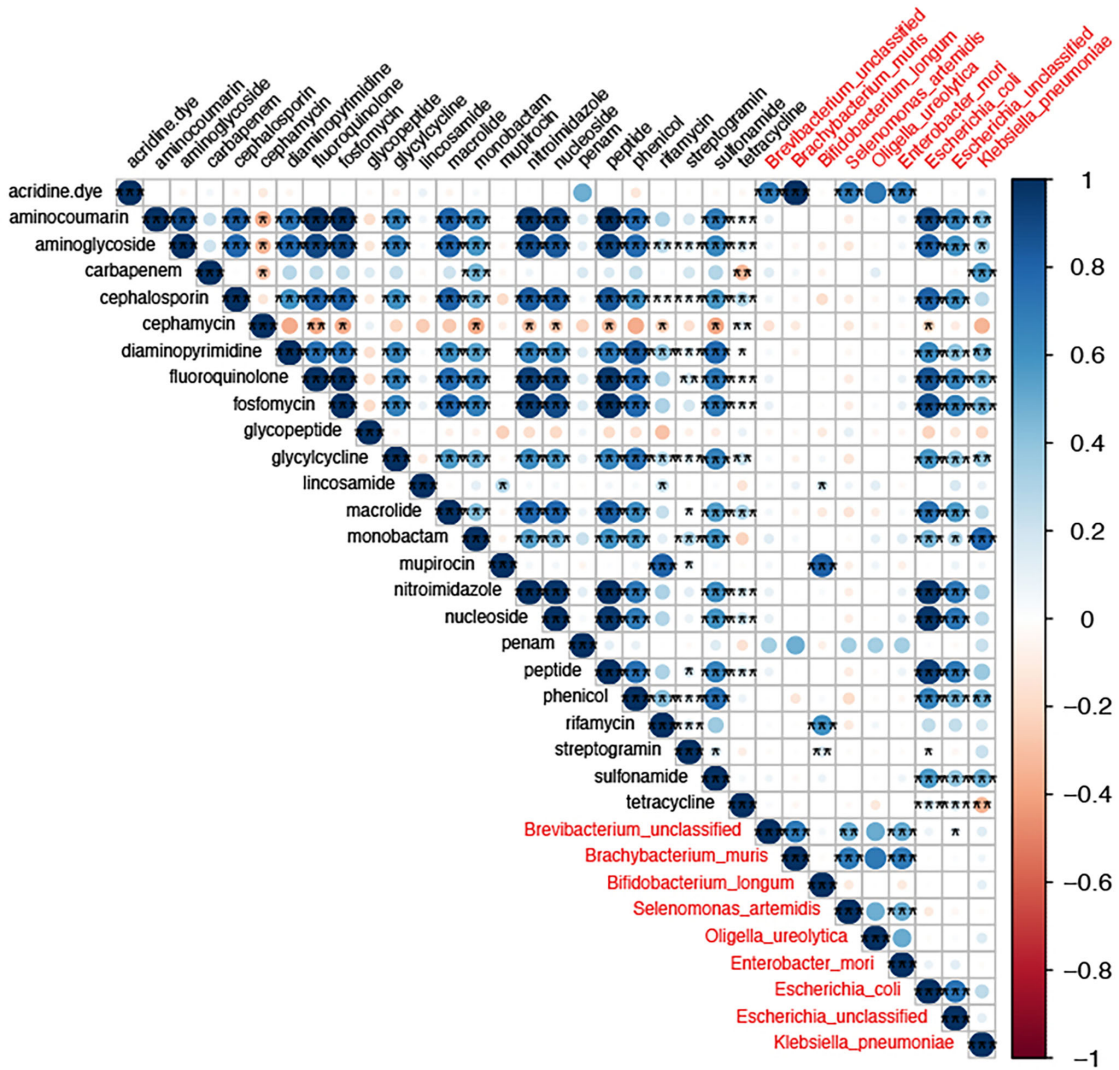
The correlation between drug resistance genes and gut strains. The size of the circle size represents the strength of the correlation and the color denotes a positive or negative correlation. The names of drug resistance genes are shown in black, and the names of bacteria are shown in red. * P-value <0.05,** P-value <0.01, and *** P-value <0.001.

To the analysis of the co-occurrence relationship between gut bacterial abundance and drug-resistant gene abundance, the threshold was set to be more than 0.5 and the p-value was less than 0.05. At the species level, nine species were highly associated with drug-resistant genes (**Figure 8**), and included *E. coli, unclassified Escherichia*, *K. pneumoniae*, and *E. mori*. Aminoglycosides, cephalosporins, quinolones, phosphomycins, macrolides, and phenol drug resistance genes were associated mainly with *E. coli*. Carbapenems, aminoglycosides, aminocoumarins, chloramphenicol, and monocyclic lactam drug resistance genes were associated mainly with *K. pneumoniae*, and rifamycin resistance genes were associated mainly with *B. longum*. The lincosamides, glycopeptides, tetracyclines, and cephalosporins were not associated significantly with any strains. Mupirocins were associated mainly with *Oligella ureolytica*. In addition, we found a strong correlation between resistance genes, further suggesting that these resistance genes may be located in the same strain. Thus, changes in the gut resistome during rifaximin-a use were associated mainly with changes in strain abundance, leading to changes in the number of resistance genes.

## Discussion

In this study, 12 weeks administration of rifaximin reduced blood ammonemia and improved neurophysiological functions in patients with cirrhosis, which is in agreement with a previous report (Bass Nathan et al., 2010). HE (West-Haven >2) occurred in five patients during the study, and except for one patient in whom excessive meat consumption was an obvious trigger for the onset of HE, all patients were post-TIPS patients with no significant changes to their usual diet. An increased incidence (30–40%) of HE has been confirmed in post-TIPS patients (Riggio et al., 2005). In addition, this study found that long-term administration of rifaximin was well tolerated, with a low incidence of general adverse events.

Stable gut microbiota diversity is of great value in maintaining liver function. Sung et al. found that the diversity of the gut microbiota decreased during onset of HE, and recovered partially during the recovery period, suggesting that changes in diversity of the gut microbiota are involved in the pathogenesis of HE (Sung et al., 2019). In this study, we also found that the diversity of the gut microbiota decreased significantly in patients who developed HE compared with those who did not, and that the decrease was most pronounced during the onset of HE. Therefore, maintaining gut microbiota diversity is important for preventing hepatic encephalopathy. Our results show that rifaximin has little effect on the diversity of the gut microbiota. In a previous cohort study, Bajaj et al. used 16s amplicon sequencing to evaluate the effect of rifaximin on the gut microbiome of patients with cirrhosis and found that rifaximin had little effect on the diversity of the gut microbiota (Kaji et al., 2017). By contrast, other antimicrobial drugs such as cefprozil, vancomycin, and ciprofloxacin decreased the number of bacterial species in the gut microbiota significantly (Raymond et al., 2016; Reijnders et al., 2016).

In addition to maintaining the diversity of gut strains, long-term administration of rifaximin may also modulate the abundance of the gut microbiota (Acharya et al., 2017). Rifaximin α slightly reduced the abundance of *Firmicutes*, and increased the abundance of *Bacteroides*, in cirrhosis patients. Patients with HE had significantly higher levels of potentially harmful strains such as *K. pneumoniae*, *K. oxytoca*, *E. faecalis*, and *C. freundii* in the intestine than patients without HE, while potentially beneficial strains such as *L. vaginalis*, *F. prausnitzii*, *A. muciniphila*, and *B. pseudocatenulatum*, and *B.breve* were significantly lower in patients with HE. These findings are in agreement with the results of previous studies (Qin et al., 2014; Llorente and Schnabl, 2015). The presence of the *Enterobacteriaceae* species is related to the initiation of inflammation in the gut tract, to the development of cirrhosis. *K. pneumoniae* and *C. freundii* are involved in the development of inflammatory bowel diseases (Henke Matthew et al., 2019), and their lipopolysaccharides can destroy the mucosa of the intestine (Song et al., 2017). We found that the abundance of *A. muciniphila* was significantly higher in patients without HE, and increased slightly during rifaximin administration. The abundance of *A. muciniphila* is negatively associated with various diseases such as IBD, obesity, acromegaly, metabolic disorders, autism, insulin resistance epilepsy, and hypertension (Everard et al., 2013; Ogata et al., 2020; Wu et al., 2020; Zhu et al., 2020). During the study period, rifaximin increased the abundance of *F. prausnitzii*, a strain that produces anti-inflammatory effects that significantly improve gut inflammation and control obesity (Sokol et al., 2008; Miquel et al., 2013). Besides, administration of rifaximin reduced the abundance of bacteria associated with intestinal barrier damage, including *Clostridium perfringens* (which has an important role in the pathogenesis of inflammatory bowel disease) (Everard et al., 2013) and *R. intestinalgutis* (which can destroy the mucosa of the intestine) (Song et al., 2017). At the same time, rifaximin maintained the abundance of probiotics. These results suggest that rifaximin may prevent HE by regulating the abundance of different strains in the gut microbiota, by reducing the abundance of harmful bacteria, and by maintaining the abundance of beneficial bacteria.

Analysis of metabolic pathways in the gut microbiota of cirrhosis patients revealed that differential activation of metabolic pathways between HE patients and non-HE patients mainly involved pathways related to cell wall synthesis and metabolism of molecules such as phosphopeptidic acid, peptidoglycan, LPS, amino acids, and fatty acids. This result indicates that these metabolic pathways may play an important role in the development of HE, although further studies are needed to confirm this. Analysis of changes in HE-related metabolic pathways revealed that those related to synthesis of aromatic amino acids, tryptophan, LPS, and branched-chain amino acids, as well as those related to the urea cycle, increased in HE patients and were either stable or decreased in non-HE patients, suggesting that metabolic changes in the gut microbiota are involved the pathogenesis of HE. We also found that branched-chain amino acid synthesis increased, suggesting that the gut microbiota compensates for increased branched-chain amino acid synthesis during HE pathogenesis. Analysis of these metabolic enhancements in conjunction with changes in the microbiota revealed that they may be mainly due to increased abundance of Enterobacteriaceae strains in the gut microbiota of cirrhosis patients. This result suggests that Enterobacteriaceae strains have an important role in promoting HE. In addition, branched-chain amino acids, aromatic ammonia, the urea cycle, and LPS-related metabolic pathways remained stable in non-HE patients, suggesting that rifaximin may prevent development of HE in cirrhotic patients by stabilizing these metabolic pathways.

The effect of long-term rifaximin administration on the genome of bacterial strains is not known. In this study, analysis of mutations in the SNV of strains showed that 12 weeks rifaximin use leads to mutations in the genome of specific strains in the gut microbiota, that these mutations lead to changes in corresponding functions, and that these functional changes alter the activity of metabolic pathways. The WSS algorithm predicted that the presence of these mutations was due to strain substitution. The main strains in which substitution occurred were *E. coli*, *B. longum*, *C. perfringens*, and *C. pratense*. After SNV occurred, the growth rate (predicted by the grid growth rate index) changed from strain to strain. Thus, under the action of rifaximin, subpopulations that were formerly dominant were gradually replaced by other subpopulations of the same species with faster growth rates. Interestingly, these substituted strains still largely maintained the abundance of the original species in the gut microbiota, further suggesting that the gut microbiota is stable, with different species forming a relatively constant proportion of the gut microbiota (D'Argenio and Salvatore, 2015). Due to the limitations of the current analysis algorithm, our results need further validation, and further studies are needed to elaborate the preventive value of the functional alterations caused by the genomic variation of the strain on the pathogenesis of HE.

Several studies show that rifaximin prevents development of HE, but the mechanism by which this occurs is not clear (Bass Nathan et al., 2010; Ito et al., 2019; Coronel-Castillo et al., 2020; Labenz et al., 2020; Lv et al., 2020). Although rifaximin exerts antibacterial effects against a wide range of Gram-positive and Gram-negative bacteria *in vitro*, our study found that rifaximin administration did not affect the stability of the gut microbiota, and that the antibacterial effect of rifaximin in the intestine was not significant. Previous studies show that rifaximin reduces plasma levels of endotoxin in patients by modulating the permeability of the gut barrier (Bajaj and Khoruts, 2020). *In vitro* studies performed in human gut epithelial cells indicate that rifaximin decreases apoptosis and increases tight junction protein expression by activating the TLR4/MyD88/NF-κB pathway, thereby increasing gut barrier function (Douhara et al., 2015; Esposito et al., 2016). The pharmacological effects of rifaximin on bacterial strains may also involve alterations in bacterial function and virulence, rather than reductions in bacterial populations (Jiang et al., 2010). Rifaximin treatment prevents HE by inhibiting bile acid production and reducing endotoxemia without altering microbiota composition (Ridlon et al., 2013). Our study suggests that rifaximin modulates gut microbiota function; the drug stabilizes gut microbiota composition and species abundance, and regulates expression of bacterial metabolic pathways through genomic variation. Through these actions, rifaximin exerts therapeutic and preventive effects on HE.

Whether long-term oral non-absorption of antimicrobial drugs leads to increased resistance is another important concern addressed by this study. We found that the greatest number of resistance genes in the gut tract of cirrhosis patients was related to penicillins, followed by tetracyclines, macrolides, and quinolones. By contrast, resistance genes in the gut microbiota of healthy people are mostly related to tetracycline antimicrobial drugs. The reason for the differences between cirrhosis patents and healthy individuals may be related to frequent hospitalization and higher frequency of antimicrobial drug use in cirrhosis patients, resulting in higher levels of resistance genes (Chaulk et al., 2014; Fernandez et al., 2016). During the 12 weeks of rifaximin administration, there was no significant increase in the total number or abundance of gut resistance genes in patients. Changes in the number of resistance genes were mostly associated with *Enterobacteriaceae* such as *E. coli* and *K. pneumoniae*, and were consistent with the process of strain changes during rifaximin administration. This finding suggests that changes in strain abundance are responsible for resistance gene changes.

A recently published study showed that patients prescribed rifaximin developed rifampin-resistant staphylococcal isolates after as little as 1-7 weeks of rifaximin treatment (Chang et al., 2017), In this study, the rifampicin resistance genes [rifampin ADP-ribosyltransferase (arr), mutations in rifamycin-resistant beta-subunit of RNA polymerase (rpoB)], were found located mainly on *B. longum or E.coli* strains. while *Staphylococcus* mainly carry the *aph*(2’), *aac(6’)*,*poxtA*, *erm*, fexA, *tetM* genes which mediate resistance to aminoglycoside, macrolide antibiotics, tetracycline respectively. However, since the previous study did not analyze the resistance mechanism, it is difficult to speculate the reason for the difference and anyway the difference between the experimental results and the sequencing results indicates that more research is needed to explore the effect of rifaximin on antibiotic resistance. In addition, no clinically important resistance genes were observed during 12 weeks use of rifaximin, and there was no significant increase in plasmids and insertion sequences related to carriage and transmission of resistance genes.

This study did not control for factors such as patient diet, lifestyle habits, and drug treatments other than antimicrobial drugs. Furthermore, differences between individuals’ life history and genetics can alter the gut microbiome. Together, these factors could influence the effect of rifaximin on the gut microbiome and drug resistance genes (Dethlefsen and Relman David, 2011). Though the number of patients included in this study was small, and many of the results were trends that did meet statistical significance, the multiple sampling and dynamic changes over time partly verify the results. Certainly, further more studies, including studies that account for patient factors, are needed to support and extend the conclusions of this study.

In conclusion,12 weeks administration of rifaximin to patients with cirrhosis is well tolerated, reduces blood ammonemia, and improves neurophysiological functions. Rifaximin does not appear to function as an antimicrobial agent in the prevention and treatment of hepatic encephalopathy, but rather as a gut microbiota regulator for the prevention of hepatic encephalopathy. Administration of rifaximin may maintain and functionally regulate the overall composition and diversity of the gut microbiota, which may reduce the abundance of harmful bacteria (e.g., *Klebsiella, Streptococcus, and Clostridium*), and increase the abundance of probiotic bacteria (e.g., *Bifidobacterium and Bacteroides*), in patients with liver cirrhosis. These changes in bacterial abundance in the gut microbiota may prevent HE by reducing the activity of metabolic pathways associated with HE attacks. 12 weeks Prophylactic administration of rifaximin in patients with cirrhosis does not alter the type and abundance of drug-resistant genes in the gut microbiota, and the number and type of drug-resistant-associated mobile elements in the gut microbiota remain stable during rifaximin administration. The induction of resistance to antimicrobial agents may not be a concern when prophylactic use this drug.

## Data Availability Statement

The datasets presented in this study can be found in online repositories. The names of the repository/repositories and accession number(s) can be found in the article/**Supplementary Material**.

## Ethics Statement

The studies involving human participants were reviewed and approved by the ethics committee of the First Affiliated Hospital, College of Medicine, Zhejiang University. The patients/participants provided their written informed consent to participate in this study.

## Author Contributions

XY, YJ, and WZ were involved in microbiome analysis, bioinformatics interpretation and research conduct. XY, ZW, JS, PS, and HG were involved in sample collection and providing clinical support. XY, BZ, QL, and TX were involved in data analysis and manuscript drafting. LL and YX conceived and designed the study. All authors contributed to the article and approved the submitted version.

## Funding

This work was supported by The National Key Research and Development Program of China (grant number 2017YFC1200203), the National Natural Science Foundation of China (grant number 81971984), the Mega-projects of Science Research of China (grant number 2018ZX10733402-004).

## Conflict of Interest

The authors declare that the research was conducted in the absence of any commercial or financial relationships that could be construed as a potential conflict of interest.

## Publisher’s Note

All claims expressed in this article are solely those of the authors and do not necessarily represent those of their affiliated organizations, or those of the publisher, the editors and the reviewers. Any product that may be evaluated in this article, or claim that may be made by its manufacturer, is not guaranteed or endorsed by the publisher.
