# Supplementary material for: Rifaximin Modulates the Gut Microbiota to Prevent Hepatic Encephalopathy in Liver Cirrhosis Without Impacting the Resistome
Source: Front Cell Infect Microbiol. 2022 Jan 18;11:761192. doi: 10.3389/fcimb.2021.761192 (PMC8804384; doi:10.3389/fcimb.2021.761192)
Supplement: Supplementary file 1 [file DataSheet_1.docx]

**Rifaximin** **modulates the gut microbiota to prevent hepatic encephalopathy in liver cirrhosis without impacting the resistome**

Xiao Yu^1,2†^, Ye Jin^1^^†^, Wangxiao Zhou^1^, Tingting Xiao^1^, Zhongwen Wu^1^, Junwei Su^1^, Hainv Gao^3^,Ping Shen^1^,Beiwen Zheng^1^, Qixia Luo^1^,Lanjuan Li^1^, Yonghong Xiao^1^

1 State Key Laboratory for Diagnosis and Treatment of Infectious Diseases, National Clinical Research Center for Infectious Diseases, First Affiliated Hospital, School of Medicine, Zhejiang University, Hangzhou, China

2 Department of Respiratory and Critical Care Medicine, First Hospital of Shanxi Medical University, Taiyuan, China

3 Department of Infectious Disease, ShuLan (Hangzhou) Hospital affiliated to Zhejiang Shuren University Shulan International Medical College, Hangzhou, China

† These authors have contributed equally to this work

**Corresponding author:** Yonghong Xiao, MD, PhD, State Key Laboratory for Diagnosis and Treatment of Infectious Diseases, National Clinical Research Center for Infectious Diseases,First Affiliated Hospital, School of Medicine, Zhejiang University, No.79 Qingchun Road, Hangzhou 310003, China Tel: 86-571-87236421; Fax: 86-571-87236421; E-mail: [xiaoyonghong@zju.edu.cn](mailto:xiaoyonghong@zju.edu.cn)

**Supplementary materials**

**Material and Methods**

**Study Design and patient enrollment**

Written informed consent was obtained from patient. The dosing regimen for rifaximin-α (trade name: Xifaxan; manufacturer: AlfaWeisman Pharma, Italy; lot number: 17567) was 400 mg three times per day, orally, for 12 weeks. During the initial observation period, patients' basic information, clinical history, and medication use were recorded. Follow-up visits were performed every 2 weeks until the end of the study, during which a digital linkage test, a digital symbol test, venous ammonia levels, routine blood tests, and liver and kidney function were measured.

The inclusion criteria were as follows: patients with cirrhosis with more than one episode of HE (Conn score, ≥2) within 6 months and in remission (Conn score, 0 or 1) at enrollment, who required rifaximin-α for prevention of HE; No history of antimicrobial agent use within three months prior to enrollment; patients aged between 18 and 70 years; patients of any gender. Exclusion criteria included allergy to rifaximin-α, clear gastrointestinal disease, inability to take the drug orally, a need for other antibacterial drugs, and patients with advanced cirrhosis who were not expected to complete the entire study cycle.

**Supplementary Figures**


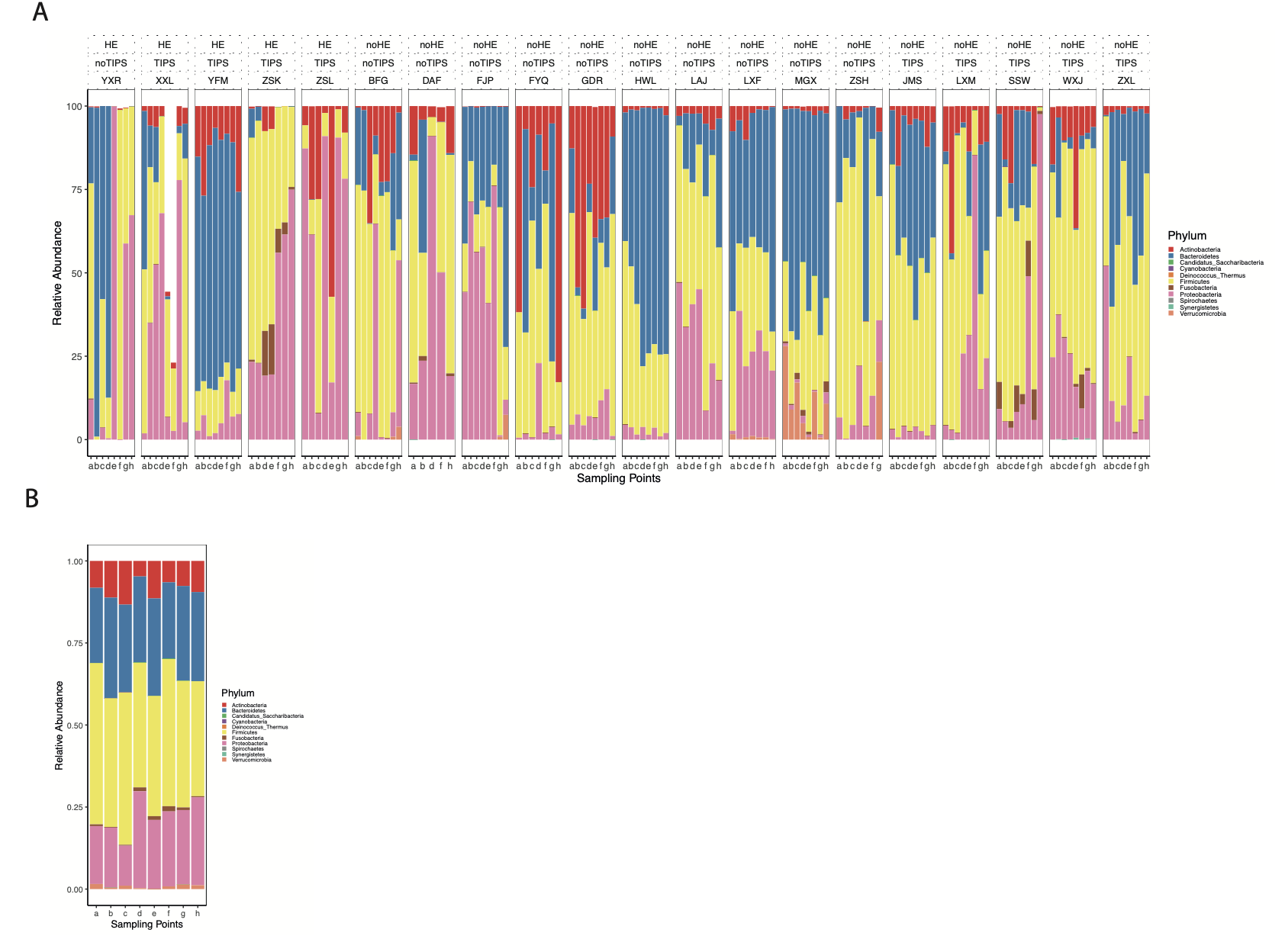


**Figure S1 Composition of gut microbiota in patients with liver cirrhosis (at a phylum level).**

Horizontal coordinates denote sampling time points; a–h represent Day 1, Week 1, Week 2, Week 4, Week 6, Week 8, Week 10, and Week 12, respectively. Vertical coordinates denote the relative abundance of the species (%). A The composition of the gut microbiota of each patient. The upper part of each diagram shows whether HE has occurred, whether it has undergone TIPS surgery and the patient's ID. B The overall composition of the gut microbiota in patients with liver cirrhosis.

A


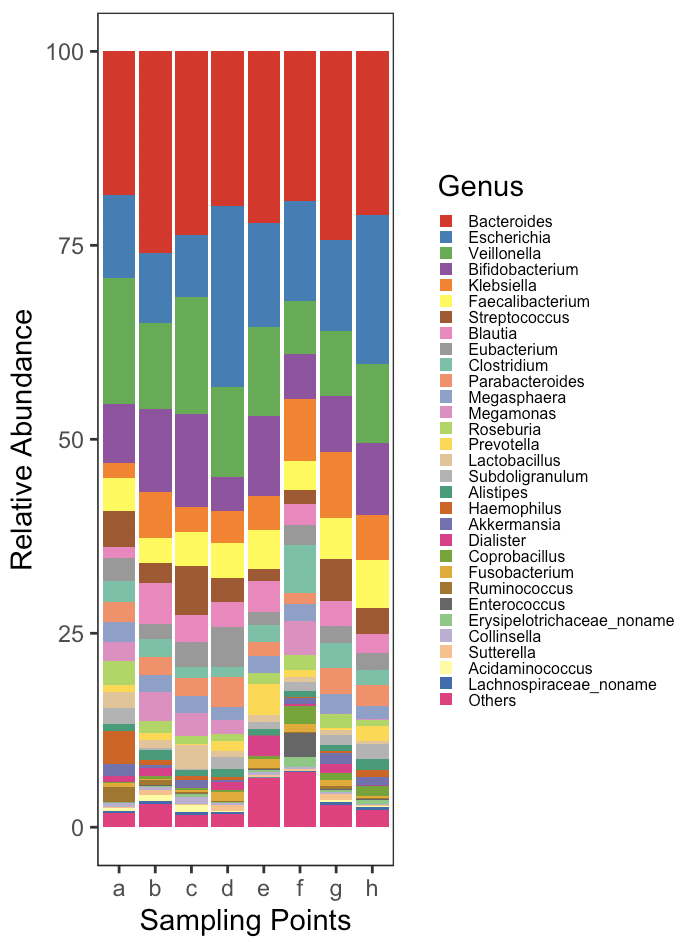


B
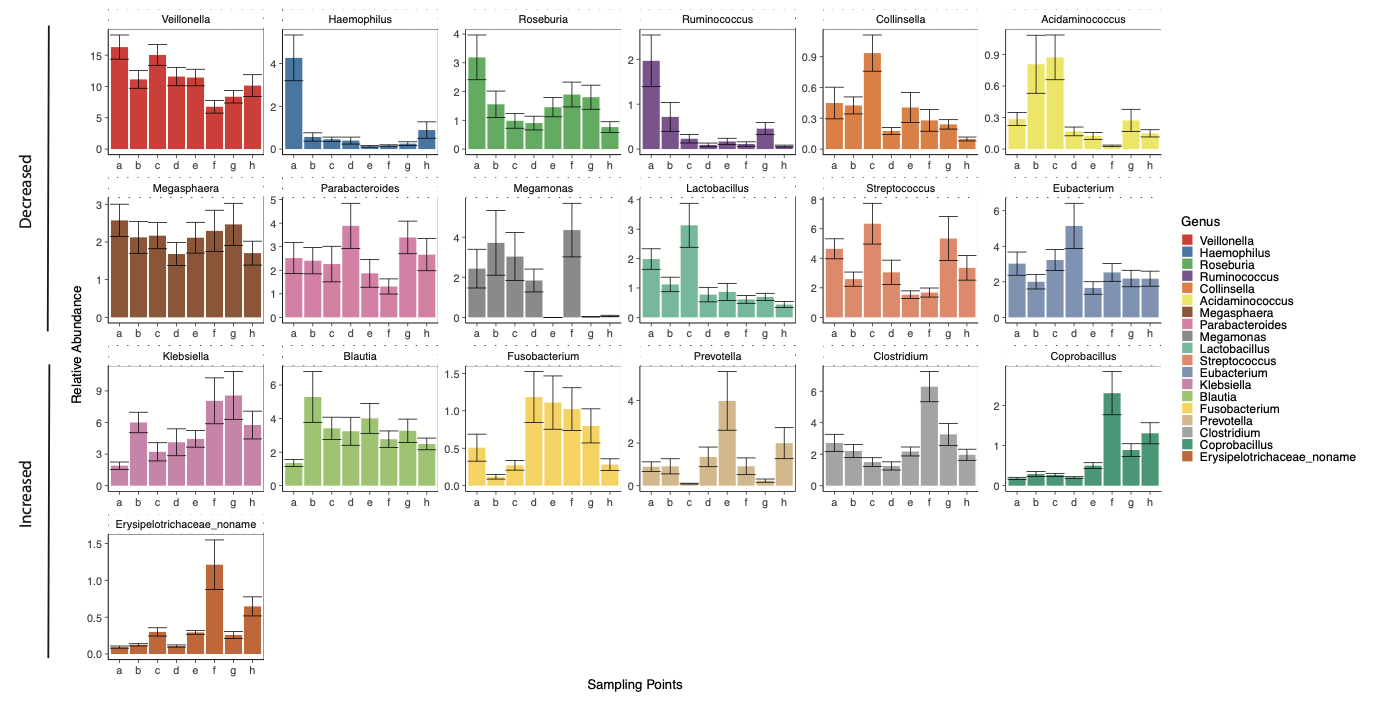


**Figure S2. Composition and** **changes in** **gut microbiota (at a genus level) in patients over time.** A The overall composition of the gut microbiota in patients with liver cirrhosis. B The gut microbiota of patients with liver cirrhosis has increased or decreased compared to Day 1. Horizontal coordinates denote sampling time points; a–h represent Day 1, Week 1, Week 2, Week 4, Week 6, Week 8, Week 10, and Week 12, respectively. Vertical coordinates denote the relative abundance of the species (%).


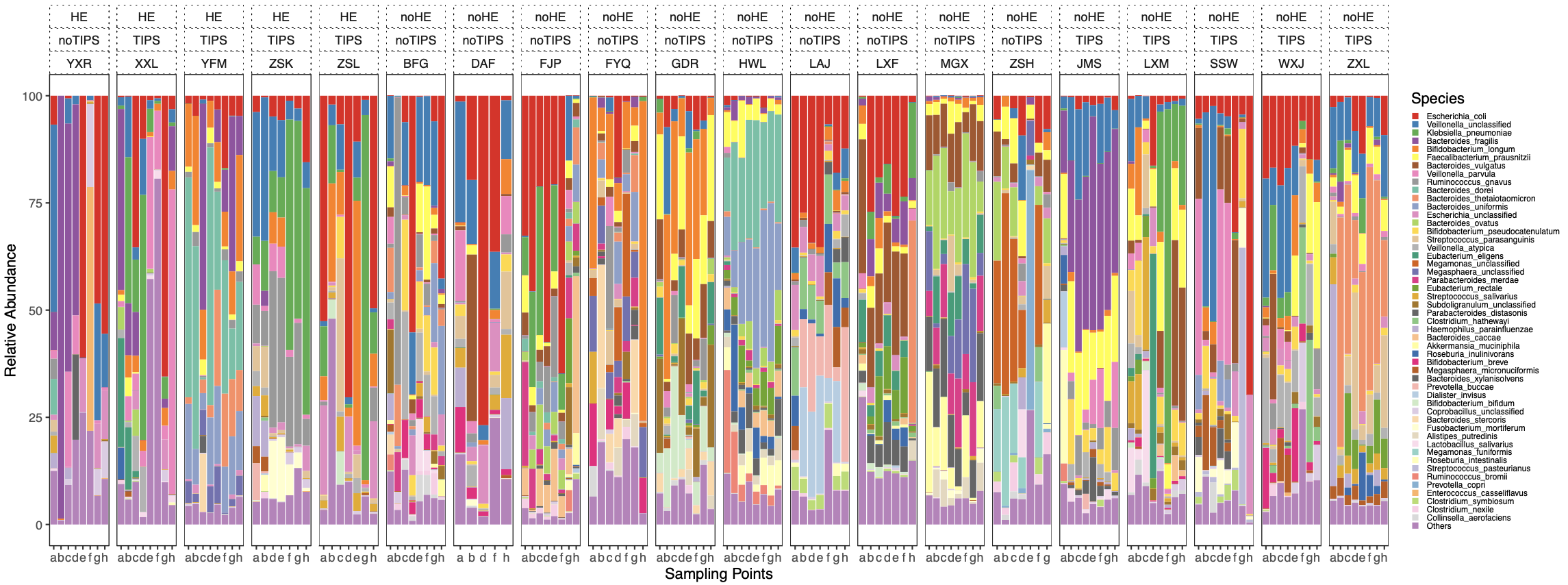


**Figure S3** **Composition of the** **gut microbiota of each patient with liver cirrhosis (at a species level)**. Horizontal coordinates denote sampling time points; a–h represent Day 1, Week 1, Week 2, Week 4, Week 6, Week 8, Week 10, and Week 12, respectively. Vertical coordinates denote the relative abundance of the species (%). A The composition diagram of the gut microbiota of each patient. The upper part of each diagram shows whether HE has occurred, whether it has undergone TIPS surgery and the patient's ID.


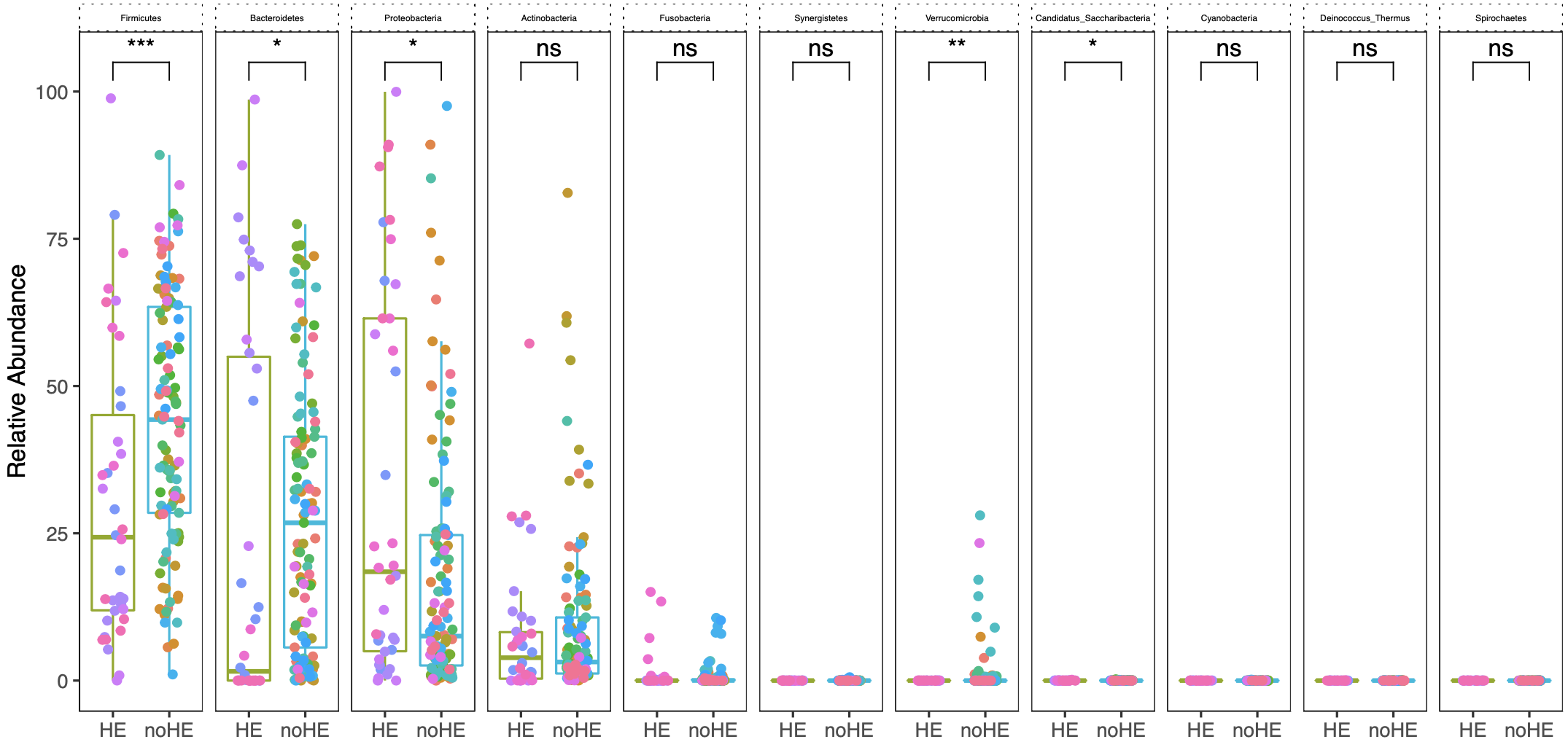


Figure S4 Differences in strain abundance (at a phylum level) between patients with HE and those without HE. Horizontal coordinates denote group(HE or no HE), and the ordinate is the relative abundance. *** means <0.001 ** means <0.01 * means <0.05 ns, no statistical difference.


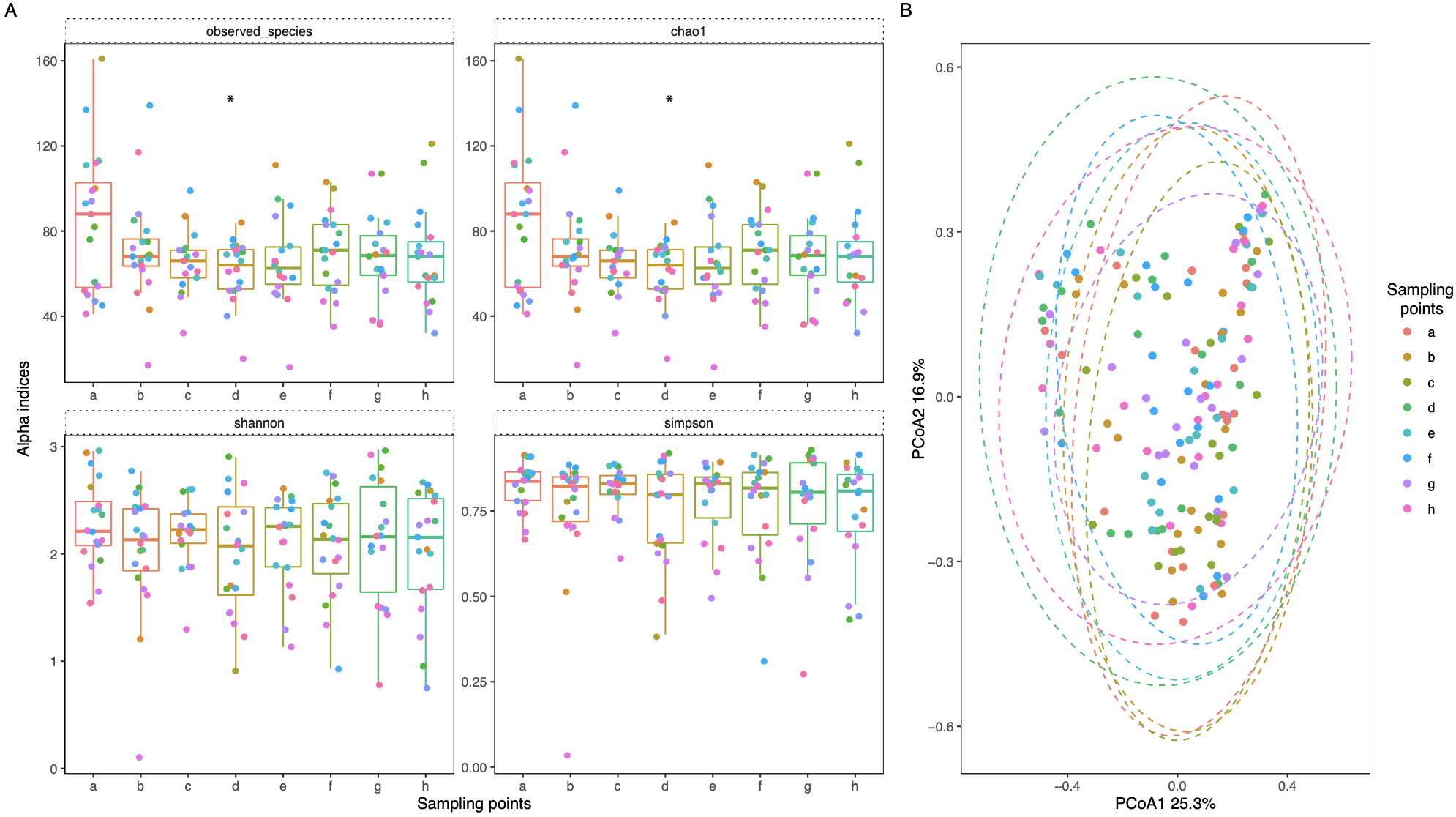


FigureS5 Diversity, composition and changes of gut microbiota in patients with liver cirrhosis. Each color point in the figure represents a patient.

A Changes in the diversity of the gut microbiota during the period of medication Horizontal coordinates denote sampling time points; a–h represent Day 1, Week 1, Week 2, Week 4, Week 6, Week 8, Week 10, and Week 12, respectively. * means that the difference is statistically significant compared with the first sampling (P<0.05). B The principal coordinate (PcoA) difference calculated based on the Bray–Curtis dissimilarity between the samples. The dashed ellipse represents the 95% confidence interval of the sample at the sampling point, and the different colors of the dashed line represent the sampling points at different times, a–h represent Day 1, Week 1, Week 2, Week 4, Week 6, Week 8, Week 10, and Week 12, respectively.


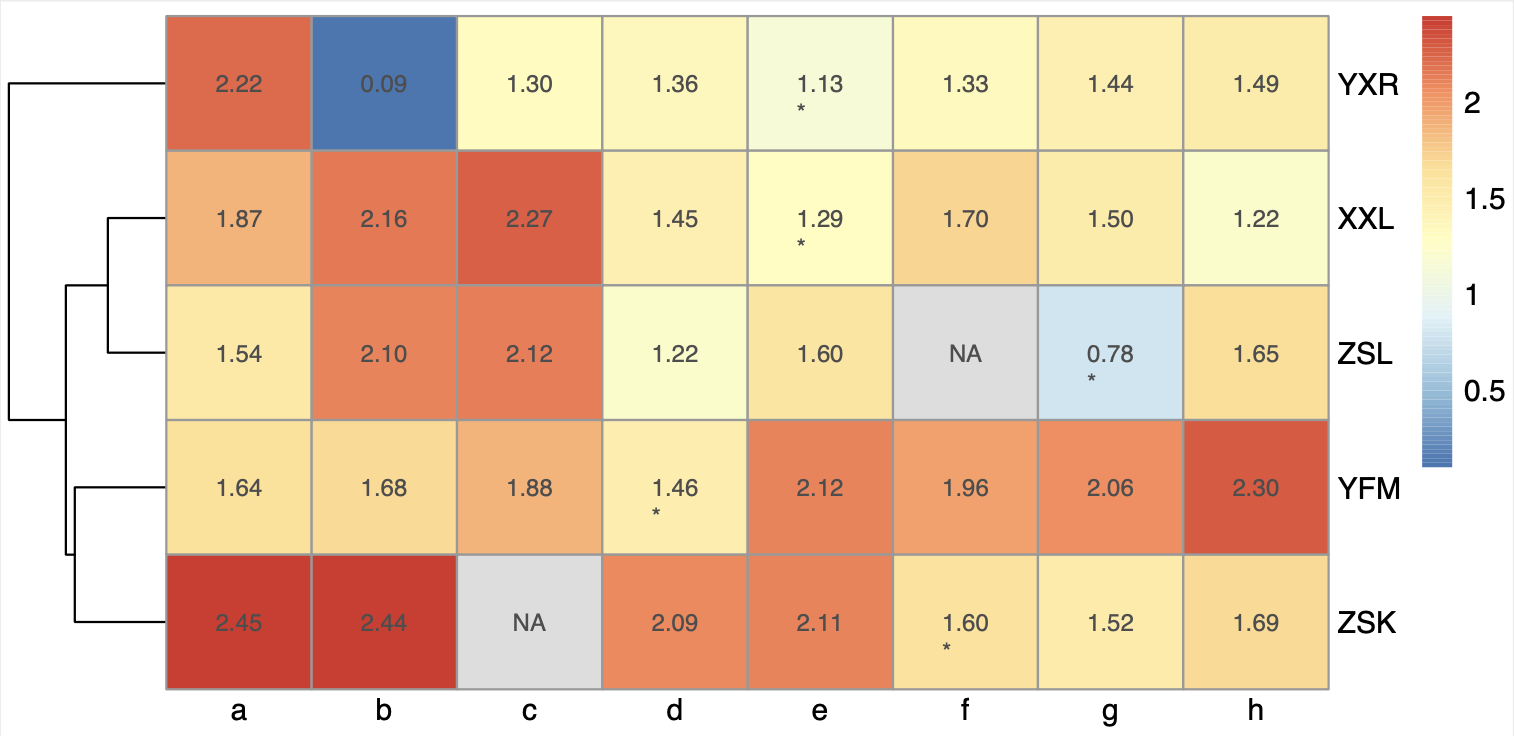


**FigureS6 Changes in** **gut microbiota diversity during the study of HE patients,** the number in the heat map is the Shannon index, and NA is not sampled. The asterisk in the heat map is the time point of the onset of HE. Horizontal coordinates denote sampling time points; a–h represent Day 1, Week 1, Week 2, Week 4, Week 6, Week 8, Week 10, and Week 12, respectively. Vertical coordinates denote the HE patient ID.


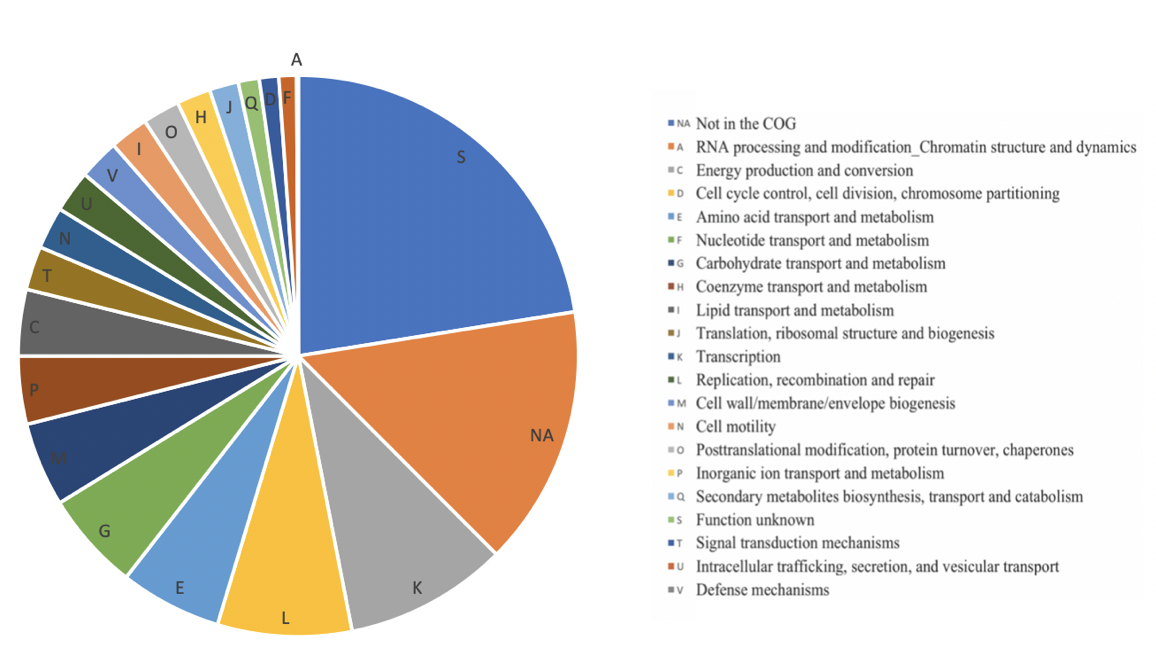


**FigureS7** COG classification of SNV genes

**
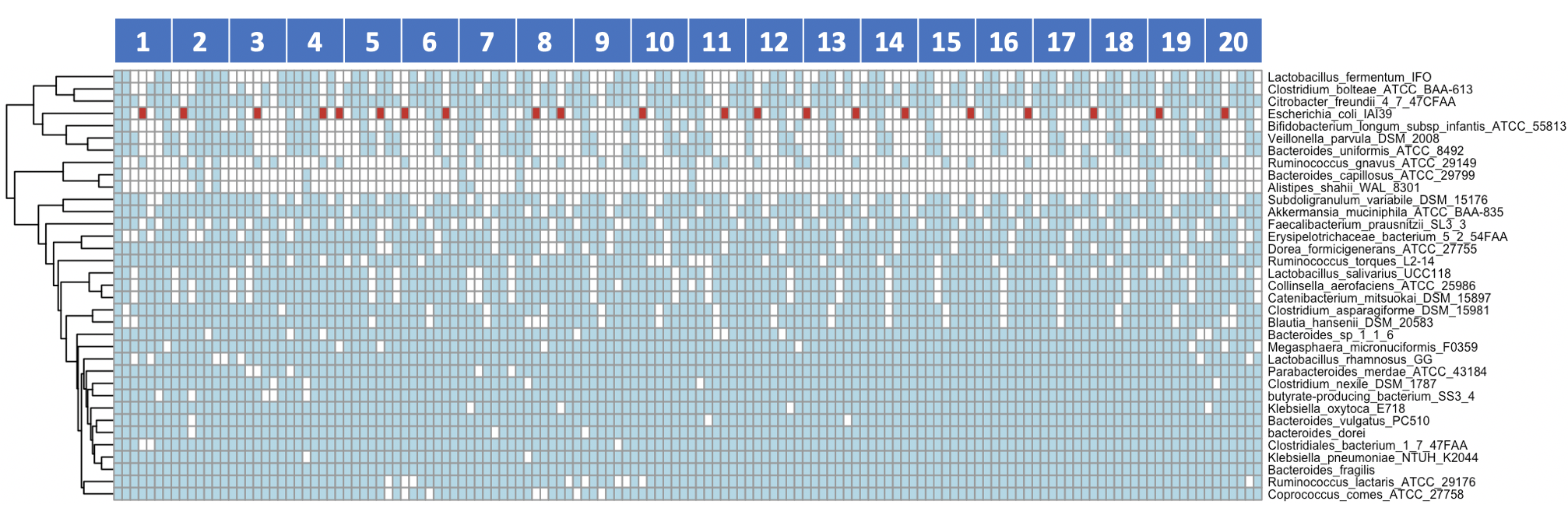
FigureS8** WSS analysis results of gut microbiota in patients with liver cirrhosis

The white in the figure represents that a new strain has replaced the original one (red represents the appearance of the third strain), and the blue represents that the strain has not been replaced. The number on the top of the heat map represents the number of each patient, and the strain name is on the right. Among them, 14, 15, 16, 18, 19 patients had hepatic encephalopathy.


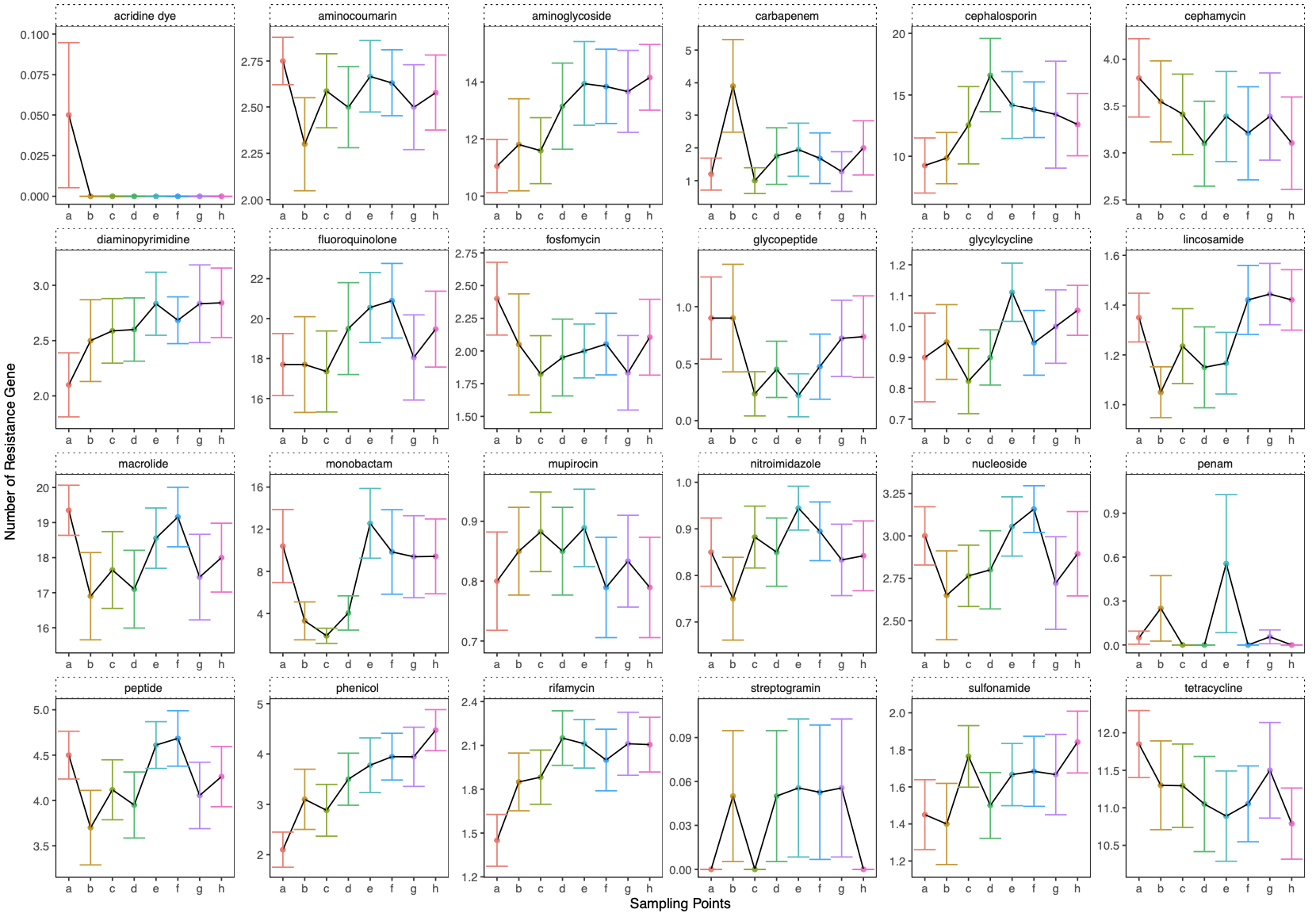


**FigureS9 Changes of 24 types of antibiotic resistance genes**

Horizontal coordinates denote sampling time points; a–h represent Day 1, Week 1, Week 2, Week 4, Week 6, Week 8, Week 10, and Week 12, respectively. Vertical coordinates denote the number of resistance genes.


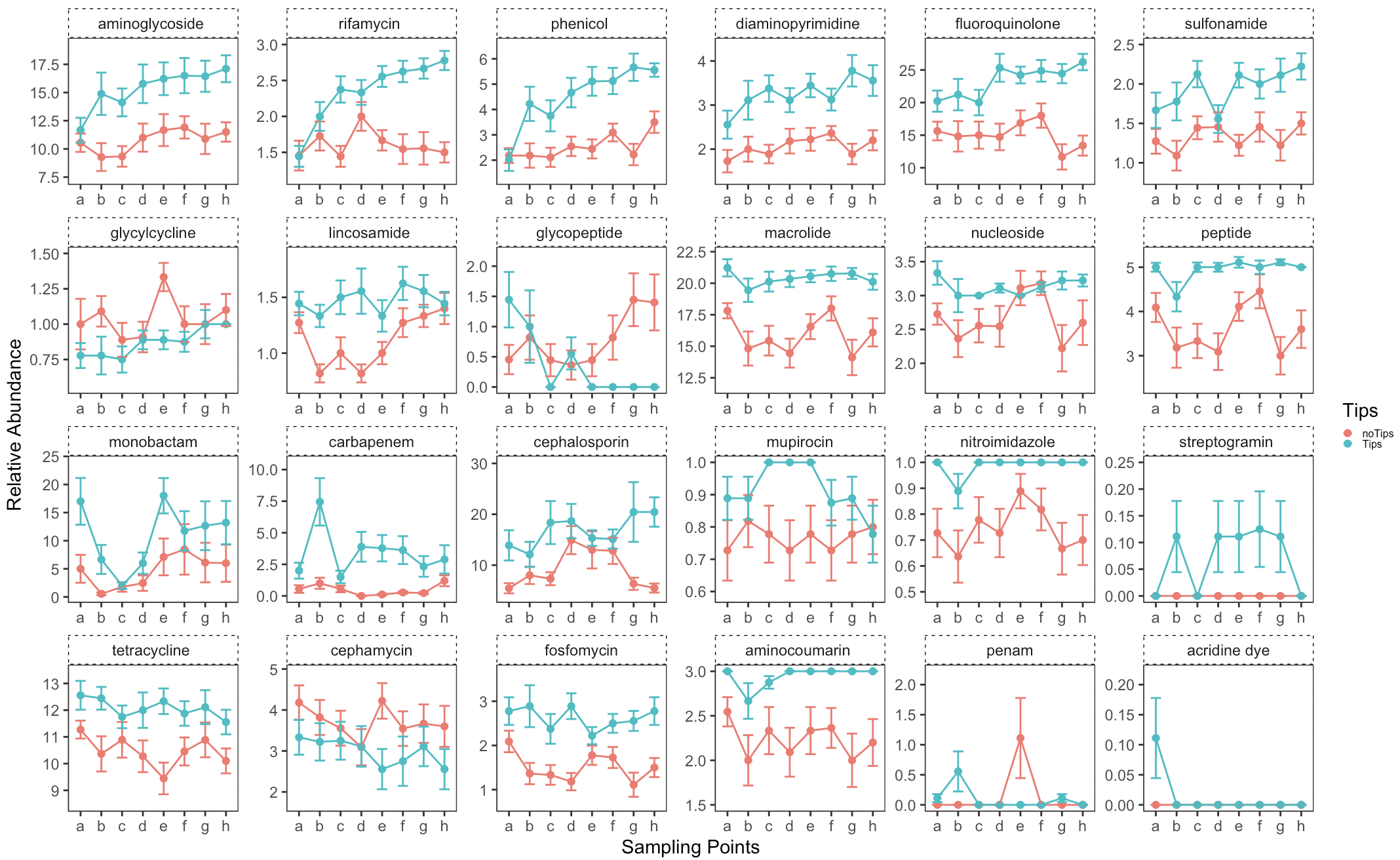


**FigureS10 Changes of 24 types of antimicrobial resistance genes in TIPS and non-TIPS patients**. Horizontal coordinates denote sampling time points; a–h represent Day 1, Week 1, Week 2, Week 4, Week 6, Week 8, Week 10, and Week 12, respectively. Vertical coordinates denote the number of resistance genes.


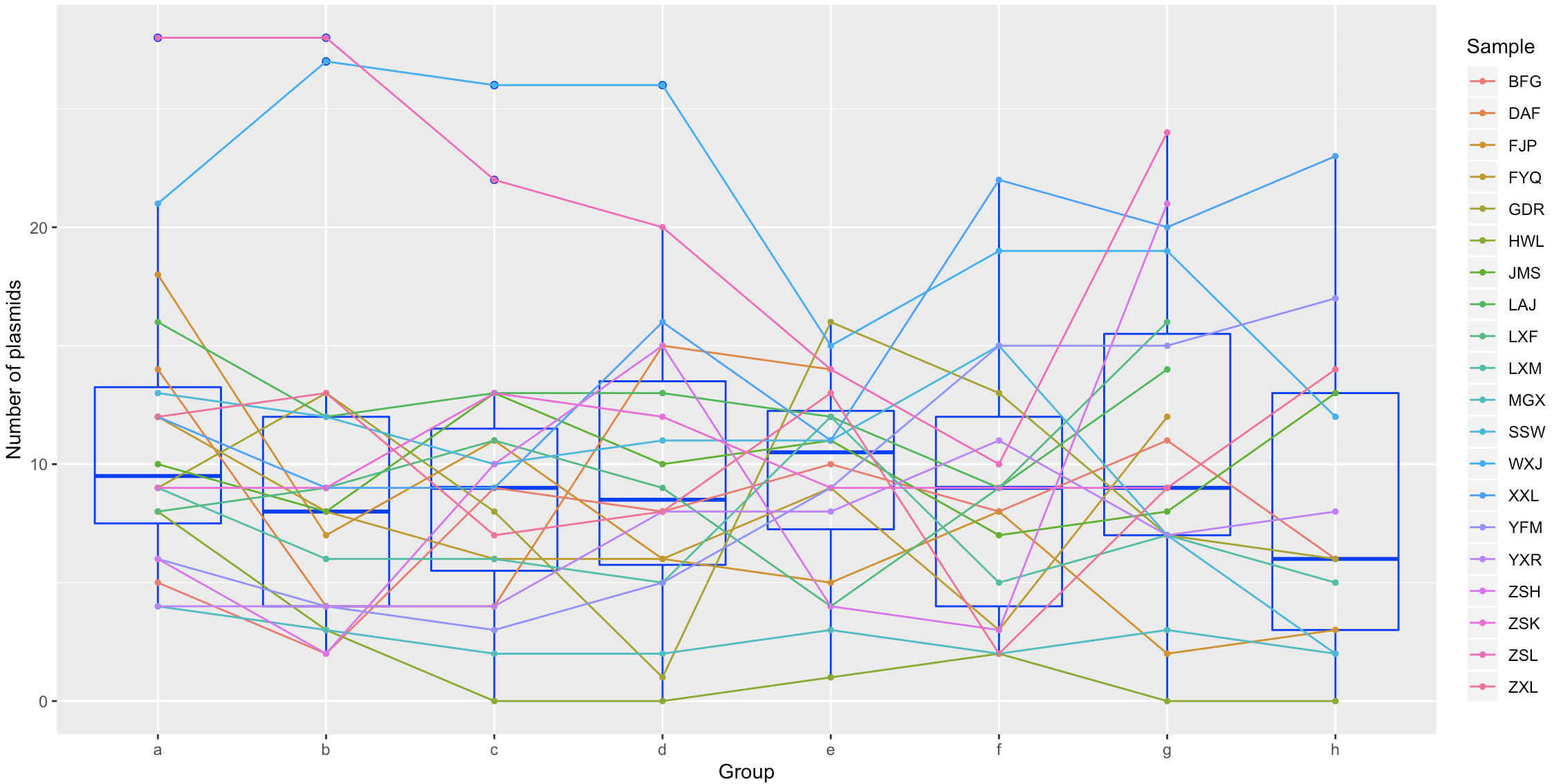


**FigureS11 Plasmid carriage in gut microbiota of patients with liver cirrhosis and its changes.**

Horizontal coordinates denote sampling time points; a–h represent Day 1, Week 1, Week 2, Week 4, Week 6, Week 8, Week 10, and Week 12, respectively. Vertical coordinates denote the number of plasmids, and the color lines represent the number of plasmids changes of each patient. The three horizontal lines of the blue box in the figure represent the upper quartile, median, and lower quartile of the number of plasmid carried by 20 patients..


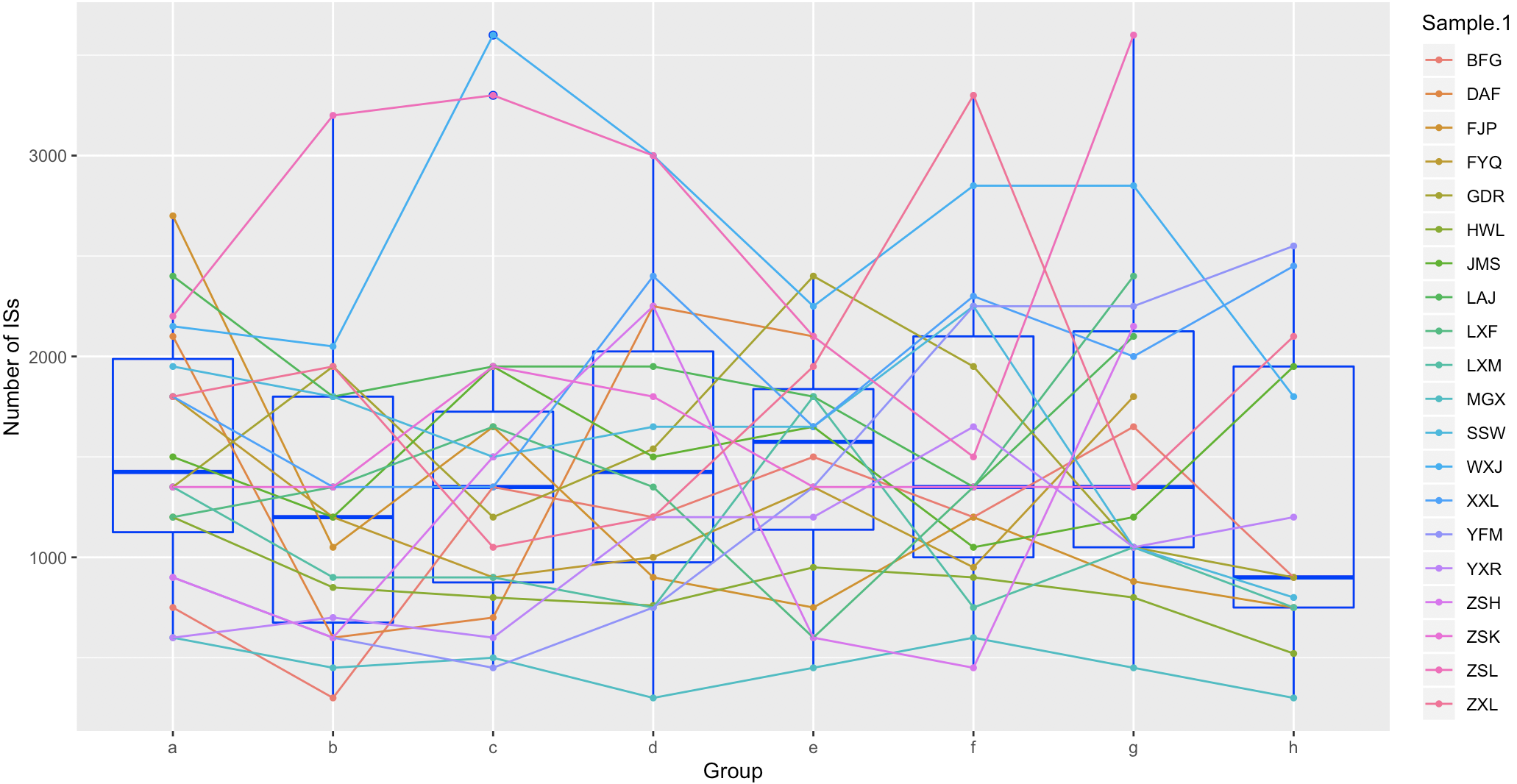


**FigureS12 IS carriage in gut microbiota of patients with liver cirrhosis and its changes.**

Horizontal coordinates denote sampling time points; a–h represent Day 1, Week 1, Week 2, Week 4, Week 6, Week 8, Week 10, and Week 12, respectively. Vertical coordinates denote the number of ISs, and the color lines represent the number of IS changes of each patient. The three horizontal lines of the blue box in the figure represent the upper quartile, median, and lower quartile of the number of ISs carried by 20 patients.

**Table S2 Results of** **physiological and biochemical test during taking Rifaximin-α**

|  | Day1 | Week1 | Week4 | Week8 | Week10 | Week12 |
| --- | --- | --- | --- | --- | --- | --- |
| **Liver function** |  |  |  |  |  |  |
| ALT(U/L) | 43.1±18.1 | 31.3 ±9.8 | 22.3±7.8 | 33.7±11.2 | 28.8±7.9 | 27.3±12.1 |
| AST(U/L) | 41.8±8.8 | 36.1±10.3 | 28.4±9.8 | 32.1±5.7 | 26.8±18.1 | 23.8±12.5 |
| TBA(μmol/L) | 49.1±6.7 | 39.8±14.6 | 45.1±10.4 | 48.3±13.1 | 23.5±7.6 | 18.1±18.1 |
| TSB(μmol/L) | 25.1±5.4 | 23.8±3.5 | 30.5±11.4 | 56.3±6.7 | 38.4±7.8 | 30.1±7.8 |
| DBIL(μmol/L) | 14.5±12.3 | 6.4±4.1 | 8.4±6.8 | 23.1±5.3 | 10.3±7.9 | 7.8±7.4 |
| IBIL(μmol/L) | 10.7±3.4 | 17.3±3.1 | 22.1±5.9 | 33.3±10.7 | 28.1±4.3 | 22.6±5.6 |
| TP(g/L) | 73.3±9.9 | 73.8±8.3 | 79.7±4.8 | 80.5±6.3 | 83.8±10.9 | 81.89±15.4 |
| Albumin (g/L) | 42.1±8.6 | 41.3±7.9 | 44.9±4.2 | 45.4±7.8 | 46.3±11.3 | 43.5±10.5 |
| Globulin (g/L) | 31.0±10.1 | 32.3±9.8 | 35.6±7.8 | 35.3±6.9 | 37.3±12.1 | 38.3±9.9 |
| **Renal function** |  |  |  |  |  |  |
| Cr(μmol/L) | 61.6±4.9 | 50.8±8.7 | 62.8±9.6 | 48.4±10.1 | 58.8±6.7 | 55.3±8.8 |
| Urea (mmol/L) | 2.7±6.4 | 4.1±7.8 | 3.6±8.3 | 4.9±9.2 | 6.2±4.3 | 7.3±5.4 |
| UA(μmol/L) | 197±78 | 320±86 | 219±73 | 109±68 | 200±69 | 150±87 |
| **Blood routine examination** |  |  |  |  |  |  |
| RBC(10E12/L) | 3.53±1.2 | 3.66±2.1 | 3.65±2.3 | 4.26±3.1 | 4.12±4.3 | 3.82±2.1 |
| Hb(g/L) | 138±23 | 122±23 | 132±17 | 142±43 | 128±21 | 130±31 |
| WBC(10E9/L) | 3.8±2.1 | 4.8±2.9 | 4.5±2.1 | 4.3±1.7 | 4.1±1.9 | 3.9±1.6 |
| PLT(10E9/L) | 89±25 | 93±21 | 120±32 | 108±25 | 98±24 | 101±31 |

AL: alanine aminotransferase, AST: aspartate aminotransferase, TBA: total biliary acid, TSB: total bilirubin, DBIL: direct bilirubin, IBIL: indirect bilirubin, TP: total protein,Cr: creatinine, UA,Uric Acid, RBC: red blood cell,Hb: hemoglobin, WBC: white blood cell,PLT: platelet

| Table S3 Differences species between the HE group and noHE group | | | | |
| --- | --- | --- | --- | --- |
| variable | HE | noHE | p | p.signif |
| Actinomyces_graevenitzii | 0.00025711 | 0.00966416 | 0.0004095 | *** |
| Actinomyces_johnsonii | 3.16E-06 | 0 | 0.0874182 | ns |
| Actinomyces_massiliensis | 0 | 6.12E-05 | 0.41678942 | ns |
| Actinomyces_odontolyticus | 0.01692974 | 0.00309239 | 0.92313635 | ns |
| Actinomyces_oris | 4.68E-05 | 1.21E-05 | 0.60786293 | ns |
| Actinomyces_sp_HPA0247 | 0 | 0.00030487 | 0.57231948 | ns |
| Actinomyces_sp_ICM47 | 0 | 0.00168496 | 0.41678942 | ns |
| Actinomyces_viscosus | 0.00013605 | 0.00010938 | 0.14079696 | ns |
| Mobiluncus_curtisii | 0 | 1.75E-05 | 0.24453154 | ns |
| Mobiluncus_unclassified | 0 | 1.44E-05 | 0.57231948 | ns |
| Varibaculum_cambriense | 0 | 7.96E-06 | 0.57231948 | ns |
| Brevibacterium_unclassified | 5.97E-05 | 2.07E-05 | 0.42932315 | ns |
| Corynebacterium_durum | 0 | 5.58E-06 | 0.57231948 | ns |
| Corynebacterium_matruchotii | 0.00015342 | 0.00012327 | 0.53753814 | ns |
| Brachybacterium_muris | 0 | 5.40E-06 | 0.57231948 | ns |
| Leifsonia_xyli | 0 | 1.21E-05 | 0.57231948 | ns |
| Leucobacter_unclassified | 0 | 3.65E-05 | 0.57231948 | ns |
| Rothia_aeria | 0.00276447 | 0.00345841 | 0.01223263 | * |
| Rothia_dentocariosa | 0.00314263 | 0.00203558 | 0.46586616 | ns |
| Rothia_mucilaginosa | 0.03179711 | 0.0351169 | 0.81862666 | ns |
| Rothia_unclassified | 0.00044921 | 0.00089009 | 0.11542837 | ns |
| Propionimicrobium_lymphophilum | 0 | 0.0002485 | 0.41678942 | ns |
| Alloscardovia_omnicolens | 0 | 0.0002046 | 0.07479878 | ns |
| Bifidobacterium_adolescentis | 4.47E-06 | 0.0025831 | 0.30088691 | ns |
| Bifidobacterium_animalis | 0.00278974 | 0.00444354 | 0.397004 | ns |
| Bifidobacterium_bifidum | 0.32671816 | 0.81000974 | 0.08999355 | ns |
| Bifidobacterium_breve | 0 | 1.11966681 | 8.77E-05 | **** |
| Bifidobacterium_catenulatum | 0 | 0.00034681 | 0.0236617 | * |
| Bifidobacterium_dentium | 0.27751184 | 0.08750504 | 0.62362486 | ns |
| Bifidobacterium_longum | 6.70822421 | 4.28639434 | 0.31334011 | ns |
| Bifidobacterium_pseudocatenulatum | 0.27443 | 2.26362885 | 3.66E-14 | **** |
| Bifidobacterium_pseudolongum | 0 | 8.74E-05 | 0.57231948 | ns |
| Gardnerella_vaginalis | 0 | 0.04516469 | 0.07479878 | ns |
| Parascardovia_denticolens | 6.39E-05 | 0.00083788 | 0.1228475 | ns |
| Scardovia_inopinata | 8.42E-05 | 0.00122381 | 0.18633065 | ns |
| Scardovia_unclassified | 0.00071158 | 0.00104867 | 0.89971736 | ns |
| Scardovia_wiggsiae | 0.00228868 | 0.00637451 | 0.2864722 | ns |
| Adlercreutzia_equolifaciens | 0 | 0.00094593 | 0.15042816 | ns |
| Atopobium_parvulum | 0.00944842 | 0.03611867 | 0.22592811 | ns |
| Atopobium_rimae | 0.00068763 | 0.00069912 | 0.45792208 | ns |
| Collinsella_aerofaciens | 0.00674 | 0.45051221 | 5.18E-07 | **** |
| Collinsella_intestinalis | 0 | 0.01546717 | 0.04723641 | * |
| Collinsella_tanakaei | 0 | 0.01795814 | 0.15042816 | ns |
| Collinsella_unclassified | 0.00949158 | 0.0032777 | 0.00771656 | ** |
| Coriobacteriaceae_bacterium_phI | 0 | 0.00407797 | 0.04723641 | * |
| Cryptobacterium_curtum | 0.0010529 | 0.02162797 | 0.91119944 | ns |
| Eggerthella_lenta | 0.00168816 | 0.00833779 | 0.78221843 | ns |
| Eggerthella_unclassified | 0.00697211 | 0.02545469 | 0.01390362 | * |
| Gordonibacter_pamelaeae | 0 | 8.12E-05 | 0.41678942 | ns |
| Olsenella_uli | 4.47E-06 | 1.15E-06 | 0.41678942 | ns |
| Olsenella_unclassified | 0.00028684 | 0.00235009 | 0.30236423 | ns |
| Slackia_exigua | 0.00113211 | 0.00866708 | 0.16310889 | ns |
| Slackia_piriformis | 0 | 0.0012023 | 0.11889073 | ns |
| Slackia_unclassified | 0.00110132 | 0.01587814 | 0.33662971 | ns |
| Bacteroides_caccae | 0.00101105 | 1.12541097 | 0.00248596 | ** |
| Bacteroides_cellulosilyticus | 0 | 0.07315434 | 0.41678942 | ns |
| Bacteroides_clarus | 0 | 0.00211177 | 0.57231948 | ns |
| Bacteroides_dorei | 6.67872053 | 1.7272154 | 0.54007531 | ns |
| Bacteroides_faecis | 0 | 8.14E-05 | 0.15042816 | ns |
| Bacteroides_fragilis | 10.6877129 | 3.07029212 | 0.17085874 | ns |
| Bacteroides_intestinalis | 0 | 0.00681602 | 0.31622332 | ns |
| Bacteroides_massiliensis | 0 | 2.23E-05 | 0.57231948 | ns |
| Bacteroides_nordii | 0.00063842 | 0.00018637 | 0.81408881 | ns |
| Bacteroides_ovatus | 0.33424132 | 2.47743894 | 0.00292705 | ** |
| Bacteroides_plebeius | 0 | 0.01545522 | 0.57231948 | ns |
| Bacteroides_salyersiae | 0 | 0.00256655 | 0.57231948 | ns |
| Bacteroides_stercoris | 0.35586 | 0.78149363 | 0.0247545 | * |
| Bacteroides_thetaiotaomicron | 1.84248868 | 3.24118221 | 0.15731489 | ns |
| Bacteroides_uniformis | 2.67102921 | 2.65113177 | 0.29616953 | ns |
| Bacteroides_vulgatus | 1.74E-05 | 5.31361044 | 5.46E-14 | **** |
| Bacteroides_xylanisolvens | 0.11005947 | 0.99694044 | 0.00265843 | ** |
| Barnesiella_intestinihominis | 0 | 0.03482673 | 0.09422328 | ns |
| Dysgonomonas_mossii | 0 | 0.00047283 | 0.57231948 | ns |
| Odoribacter_splanchnicus | 0 | 0.01892628 | 0.09422328 | ns |
| Parabacteroides_distasonis | 0.52330237 | 1.17890204 | 2.94E-05 | **** |
| Parabacteroides_goldsteinii | 0 | 0.00628823 | 0.02981505 | * |
| Parabacteroides_johnsonii | 0 | 0.02741664 | 0.01875818 | * |
| Parabacteroides_merdae | 0 | 1.68071186 | 1.50E-05 | **** |
| Parabacteroides_unclassified | 0.48352053 | 0.1725862 | 0.01271106 | * |
| Porphyromonas_asaccharolytica | 0.00011974 | 0.00999053 | 0.65598852 | ns |
| Porphyromonas_bennonis | 0.00024368 | 0.00586885 | 0.98352253 | ns |
| Porphyromonas_endodontalis | 0 | 1.89E-05 | 0.57231948 | ns |
| Porphyromonas_gingivalis | 0 | 4.51E-06 | 0.57231948 | ns |
| Porphyromonas_somerae | 0 | 0.0101362 | 0.15042816 | ns |
| Porphyromonas_uenonis | 0 | 0.00400602 | 0.07479878 | ns |
| Alloprevotella_rava | 0 | 3.36E-05 | 0.57231948 | ns |
| Alloprevotella_tannerae | 0.00039974 | 0.00034381 | 0.01813182 | * |
| Alloprevotella_unclassified | 5.11E-05 | 5.61E-05 | 0.85524856 | ns |
| Paraprevotella_clara | 0 | 0.00471549 | 0.19115076 | ns |
| Paraprevotella_unclassified | 0 | 0.03996743 | 0.19115076 | ns |
| Candidatus_Prevotella_conceptionensis | 0 | 7.57E-05 | 0.41678942 | ns |
| Prevotella_baroniae | 0 | 0.00001 | 0.57231948 | ns |
| Prevotella_bivia | 0 | 0.04055858 | 0.00926739 | ** |
| Prevotella_buccae | 0.23953763 | 0.91989354 | 0.10320006 | ns |
| Prevotella_buccalis | 3.68E-06 | 0.00046743 | 0.19558475 | ns |
| Prevotella_copri | 0 | 0.52265708 | 0.11889073 | ns |
| Prevotella_dentalis | 0.00014237 | 8.04E-05 | 0.74949859 | ns |
| Prevotella_denticola | 0.14660263 | 0.00089115 | 0.0169286 | * |
| Prevotella_disiens | 0 | 0.00159266 | 0.11889073 | ns |
| Prevotella_histicola | 0.00019737 | 0.01242867 | 0.02670704 | * |
| Prevotella_intermedia | 0 | 0.0009377 | 0.57231948 | ns |
| Prevotella_loescheii | 0.00011711 | 0 | 0.01485021 | * |
| Prevotella_melaninogenica | 0.00016921 | 0.00180266 | 0.92418438 | ns |
| Prevotella_multiformis | 0 | 0.00034522 | 0.57231948 | ns |
| Prevotella_multisaccharivorax | 0 | 0.00751708 | 0.11889073 | ns |
| Prevotella_nanceiensis | 0 | 0.00043345 | 0.31622332 | ns |
| Prevotella_nigrescens | 0.00014553 | 0.00105982 | 0.66203329 | ns |
| Prevotella_oralis | 0 | 0.00028549 | 0.31622332 | ns |
| Prevotella_oris | 0.00112132 | 0.05672496 | 0.66203329 | ns |
| Prevotella_oulorum | 4.16E-05 | 0.00028327 | 0.77144524 | ns |
| Prevotella_pallens | 0.00081579 | 0.00036177 | 0.80934364 | ns |
| Prevotella_pleuritidis | 0 | 1.58E-05 | 0.57231948 | ns |
| Prevotella_salivae | 0.00018395 | 0.00060221 | 0.25889711 | ns |
| Prevotella_stercorea | 0 | 0.02156319 | 0.57231948 | ns |
| Prevotella_timonensis | 0.00075711 | 0.00842487 | 0.33710151 | ns |
| Prevotella_veroralis | 0.00015342 | 0.00147336 | 0.50231211 | ns |
| Alistipes_finegoldii | 0 | 0.01548133 | 0.01875818 | * |
| Alistipes_indistinctus | 0 | 4.81E-05 | 0.41678942 | ns |
| Alistipes_onderdonkii | 0 | 0.05283991 | 0.19115076 | ns |
| Alistipes_putredinis | 0 | 0.79223133 | 0.00077405 | *** |
| Alistipes_senegalensis | 0 | 0.0002031 | 0.57231948 | ns |
| Alistipes_shahii | 0 | 0.07631876 | 0.0148517 | * |
| Alistipes_sp_AP11 | 0 | 0.06523974 | 0.11889073 | ns |
| Alistipes_unclassified | 0 | 0.29393797 | 0.03753781 | * |
| Capnocytophaga_gingivalis | 0 | 8.98E-05 | 0.57231948 | ns |
| Capnocytophaga_granulosa | 0 | 2.39E-06 | 0.57231948 | ns |
| Capnocytophaga_unclassified | 0 | 0.00026841 | 0.15042816 | ns |
| Chryseobacterium_unclassified | 0 | 4.29E-05 | 0.41678942 | ns |
| Empedobacter_brevis | 0 | 1.58E-05 | 0.57231948 | ns |
| Myroides_unclassified | 0 | 8.94E-06 | 0.57231948 | ns |
| Riemerella_unclassified | 0 | 0.00057062 | 0.31622332 | ns |
| Sphingobacterium_unclassified | 0 | 0.00016469 | 0.15042816 | ns |
| candidate_division_TM7_single_cell_isolate_TM7b | 0.00167263 | 0.00376974 | 0.07383998 | ns |
| candidate_division_TM7_single_cell_isolate_TM7c | 0.00274526 | 0.00733664 | 0.03198551 | * |
| Arthrospira_maxima | 0 | 0.00037018 | 0.41678942 | ns |
| Arthrospira_platensis | 0 | 0.00039823 | 0.41678942 | ns |
| Arthrospira_unclassified | 0 | 0.00150487 | 0.41678942 | ns |
| Deinococcus_unclassified | 0 | 0.00011133 | 0.31622332 | ns |
| Anoxybacillus_flavithermus | 0 | 2.51E-05 | 0.57231948 | ns |
| Anoxybacillus_unclassified | 0 | 3.31E-05 | 0.41678942 | ns |
| Bacillus_licheniformis | 0 | 2.65E-06 | 0.57231948 | ns |
| Geobacillus_unclassified | 0 | 1.81E-05 | 0.57231948 | ns |
| Gemella_haemolysans | 0.00168579 | 0.01361177 | 0.09848085 | ns |
| Gemella_morbillorum | 0.00067684 | 0.00098566 | 0.06357752 | ns |
| Gemella_sanguinis | 0.00549026 | 0.00613496 | 0.08784865 | ns |
| Gemella_unclassified | 0.0003379 | 0.00066345 | 0.03876389 | * |
| Staphylococcus_aureus | 0 | 0.00043018 | 0.15042816 | ns |
| Staphylococcus_epidermidis | 3.92E-05 | 2.12E-05 | 0.42932315 | ns |
| Abiotrophia_defectiva | 0.00062026 | 0.00030053 | 0.88649051 | ns |
| Granulicatella_adiacens | 0.01279211 | 0.01958655 | 0.29154865 | ns |
| Granulicatella_elegans | 0.00125763 | 0.00022274 | 0.00033937 | *** |
| Granulicatella_unclassified | 0.02251711 | 0.03719009 | 0.28822988 | ns |
| Enterococcus_asini | 7.37E-06 | 0.00020487 | 0.76298624 | ns |
| Enterococcus_avium | 0.04183763 | 0.03441805 | 0.89525164 | ns |
| Enterococcus_casseliflavus | 1.508645 | 0.0100208 | 0.00968744 | ** |
| Enterococcus_dispar | 0 | 0.00010212 | 0.31622332 | ns |
| Enterococcus_durans | 0.00141737 | 0 | 3.34E-06 | **** |
| Enterococcus_faecalis | 0.00027579 | 0.00209035 | 0.00490987 | ** |
| Enterococcus_faecium | 0.03734526 | 0.00625876 | 0.00020151 | *** |
| Enterococcus_gallinarum | 0.05659316 | 0.00035788 | 2.88E-05 | **** |
| Enterococcus_hirae | 0 | 5.47E-05 | 0.57231948 | ns |
| Enterococcus_raffinosus | 0.01266237 | 1.06E-05 | 1.27E-10 | **** |
| Lactobacillus_acidophilus | 0.00159316 | 0.0077054 | 0.62570087 | ns |
| Lactobacillus_amylovorus | 0.02167342 | 1.18E-05 | 0.00014253 | *** |
| Lactobacillus_casei_paracasei | 0.13614158 | 0.03491451 | 1.60E-09 | **** |
| Lactobacillus_crispatus | 0.00788342 | 0.16706327 | 0.00016199 | *** |
| Lactobacillus_delbrueckii | 0.21369947 | 0.00430965 | 0.12100945 | ns |
| Lactobacillus_equicursoris | 0.00069342 | 0 | 0.00050935 | *** |
| Lactobacillus_fermentum | 0.23361342 | 0.19106735 | 0.00644562 | ** |
| Lactobacillus_gasseri | 0.01443395 | 0.00684788 | 0.44762894 | ns |
| Lactobacillus_helveticus | 0.00284947 | 0 | 0.0874182 | ns |
| Lactobacillus_johnsonii | 0 | 0.03310177 | 0.57231948 | ns |
| Lactobacillus_mucosae | 0.02199342 | 0.03215053 | 0.00978396 | ** |
| Lactobacillus_oris | 0.00023158 | 0.00114372 | 0.37101444 | ns |
| Lactobacillus_pentosus | 0 | 3.63E-06 | 0.57231948 | ns |
| Lactobacillus_plantarum | 0.00086105 | 0.00093991 | 0.72399557 | ns |
| Lactobacillus_reuteri | 0 | 0.00196956 | 0.19115076 | ns |
| Lactobacillus_rhamnosus | 0.03878816 | 0.00434195 | 5.87E-11 | **** |
| Lactobacillus_salivarius | 0.17396974 | 0.6915069 | 0.11532838 | ns |
| Lactobacillus_sanfranciscensis | 0.00800895 | 0.05956425 | 0.48431235 | ns |
| Lactobacillus_vaginalis | 0 | 0.05043443 | 0.00275715 | ** |
| Pediococcus_pentosaceus | 1.13E-05 | 0 | 0.0874182 | ns |
| Leuconostoc_citreum | 0.00122316 | 0.00129425 | 0.67956384 | ns |
| Leuconostoc_lactis | 0.00469974 | 0.00494266 | 0.34201184 | ns |
| Leuconostoc_mesenteroides | 0 | 0.00012 | 0.57231948 | ns |
| Leuconostoc_pseudomesenteroides | 0 | 3.19E-06 | 0.41678942 | ns |
| Leuconostoc_unclassified | 0.00031 | 0.00012283 | 0.05679573 | ns |
| Weissella_cibaria | 0.00032316 | 0.00063885 | 0.99385088 | ns |
| Weissella_confusa | 0.01344868 | 0.00514912 | 0.00821386 | ** |
| Weissella_unclassified | 6.47E-05 | 0.00041496 | 0.62570087 | ns |
| Lactococcus_garvieae | 0.058735 | 0.00129487 | 0.42321065 | ns |
| Lactococcus_lactis | 0.02262263 | 0.00179292 | 0.42363983 | ns |
| Lactococcus_raffinolactis | 0 | 0.0001777 | 0.57231948 | ns |
| Streptococcus_anginosus | 0.00893211 | 0.01154195 | 0.10015509 | ns |
| Streptococcus_australis | 0.00746684 | 0.00397982 | 0.06504573 | ns |
| Streptococcus_constellatus | 0.00012421 | 6.65E-05 | 0.18274405 | ns |
| Streptococcus_cristatus | 0.00297158 | 0.00172434 | 0.84610767 | ns |
| Streptococcus_gordonii | 0.01783395 | 0.00814611 | 0.7139972 | ns |
| Streptococcus_infantarius | 0.00015974 | 5.64E-05 | 0.81408881 | ns |
| Streptococcus_infantis | 0.01885184 | 0.00505743 | 0.2269391 | ns |
| Streptococcus_intermedius | 0.00046868 | 0.00117513 | 0.5353747 | ns |
| Streptococcus_lutetiensis | 0 | 0.00034805 | 0.31622332 | ns |
| Streptococcus_mitis_oralis_pneumoniae | 0.07420105 | 0.01816805 | 0.00976427 | ** |
| Streptococcus_mutans | 0.01735368 | 0.00552327 | 0.12168604 | ns |
| Streptococcus_oligofermentans | 0.00068026 | 0.00049478 | 0.99679799 | ns |
| Streptococcus_parasanguinis | 1.76383158 | 1.78211354 | 0.06743071 | ns |
| Streptococcus_pasteurianus | 0.00913 | 0.57505478 | 0.22617874 | ns |
| Streptococcus_peroris | 0.00021158 | 0.00025089 | 0.88505004 | ns |
| Streptococcus_pseudopneumoniae | 0.00064105 | 0.00012336 | 0.19767025 | ns |
| Streptococcus_salivarius | 0.832945 | 1.25954929 | 0.11115851 | ns |
| Streptococcus_sanguinis | 0.02623132 | 0.02410133 | 0.15977334 | ns |
| Streptococcus_sobrinus | 0.00165079 | 0.00130469 | 0.09641612 | ns |
| Streptococcus_sp_BS35b | 0.00028737 | 0.00053345 | 0.78204705 | ns |
| Streptococcus_thermophilus | 0.00191526 | 0.02258708 | 0.74800588 | ns |
| Streptococcus_tigurinus | 0.00310421 | 0.00221903 | 0.68746344 | ns |
| Streptococcus_vestibularis | 0.04986105 | 0.03411637 | 0.8044263 | ns |
| Butyricicoccus_pullicaecorum | 0.00017763 | 0.0076623 | 0.25889711 | ns |
| Clostridiaceae_bacterium_JC118 | 0 | 0.00976743 | 0.03753781 | * |
| Clostridium_asparagiforme | 0 | 0.03364372 | 0.00129653 | ** |
| Clostridium_bolteae | 0.02001974 | 0.45226089 | 3.23E-06 | **** |
| Clostridium_butyricum | 0.38381605 | 0.20007805 | 0.19011321 | ns |
| Clostridium_celatum | 0.00432974 | 0.00735717 | 0.74949859 | ns |
| Clostridium_citroniae | 0 | 0.08892876 | 0.00011635 | *** |
| Clostridium_clostridioforme | 0.01221026 | 0.24498735 | 0.00136732 | ** |
| Clostridium_hathewayi | 0.02015763 | 1.26121549 | 7.49E-07 | **** |
| Clostridium_leptum | 0 | 0.00017735 | 0.31622332 | ns |
| Clostridium_methylpentosum | 0 | 0.00092044 | 0.41678942 | ns |
| Clostridium_nexile | 0.05958947 | 0.44724469 | 4.51E-05 | **** |
| Clostridium_perfringens | 0.16538737 | 0.10120239 | 0.03421478 | * |
| Clostridium_scindens | 0 | 3.96E-05 | 0.31622332 | ns |
| Clostridium_sp_7_2_43FAA | 0.05524579 | 0.00207106 | 6.51E-06 | **** |
| Clostridium_sp_D5 | 0 | 0.0023754 | 0.24453154 | ns |
| Clostridium_sp_HGF2 | 0 | 0.00629044 | 0.41678942 | ns |
| Clostridium_symbiosum | 0.01057105 | 0.47658425 | 0.00199816 | ** |
| Eubacterium_infirmum | 0.00020263 | 0.00022035 | 0.54452542 | ns |
| Mogibacterium_sp_CM50 | 0.00165605 | 0 | 0.0874182 | ns |
| Anaerococcus_tetradius | 0 | 0.00036407 | 0.31622332 | ns |
| Anaerococcus_vaginalis | 0 | 1.43E-05 | 0.57231948 | ns |
| Clostridiales_bacterium_BV3C26 | 0 | 0.00045823 | 0.15042816 | ns |
| Finegoldia_magna | 0 | 0.00014354 | 0.19115076 | ns |
| Parvimonas_micra | 0 | 5.66E-06 | 0.41678942 | ns |
| Parvimonas_unclassified | 0 | 1.76E-05 | 0.57231948 | ns |
| Peptoniphilus_duerdenii | 0 | 8.17E-05 | 0.31622332 | ns |
| Peptoniphilus_harei | 0 | 0.00129779 | 0.31622332 | ns |
| Peptoniphilus_lacrimalis | 1.42E-05 | 0.00195531 | 0.15513578 | ns |
| Clostridiales_bacterium_1_7_47FAA | 0.00012579 | 0.04176974 | 6.78E-06 | **** |
| Flavonifractor_plautii | 0.09296842 | 0.10003 | 0.0704202 | ns |
| Anaerofustis_stercorihominis | 0 | 0.00032858 | 0.19115076 | ns |
| Eubacterium_brachy | 0 | 5.31E-05 | 0.31622332 | ns |
| Eubacterium_eligens | 1.09230579 | 1.73432575 | 0.04208458 | * |
| Eubacterium_hallii | 0 | 0.01217841 | 0.00275721 | ** |
| Eubacterium_limosum | 2.87E-05 | 0.00116788 | 0.31576967 | ns |
| Eubacterium_ramulus | 0 | 0.00937646 | 0.09422328 | ns |
| Eubacterium_rectale | 0.00354579 | 1.53079726 | 0.00098164 | *** |
| Eubacterium_saphenum | 0 | 0.0001477 | 0.31622332 | ns |
| Eubacterium_siraeum | 0 | 0.0013631 | 0.41678942 | ns |
| Eubacterium_sp_3_1_31 | 0 | 0.00055699 | 0.15042816 | ns |
| Eubacterium_ventriosum | 0.0053329 | 0.02912239 | 0.39194496 | ns |
| Pseudoramibacter_alactolyticus | 0 | 0.00025451 | 0.57231948 | ns |
| Anaerostipes_caccae | 2.24E-05 | 0.00011071 | 0.61675363 | ns |
| Anaerostipes_hadrus | 0.0061229 | 0.08717788 | 0.03589119 | * |
| Anaerostipes_unclassified | 0.00079237 | 0.0106708 | 0.02053158 | * |
| Blautia_hydrogenotrophica | 0 | 7.70E-05 | 0.57231948 | ns |
| Blautia_producta | 4.63E-05 | 0.00382717 | 0.0631572 | ns |
| Ruminococcus_gnavus | 4.47508737 | 2.47250699 | 0.27264644 | ns |
| Ruminococcus_obeum | 0.00835237 | 0.04263522 | 1.35E-06 | **** |
| Ruminococcus_torques | 0.09546316 | 0.24928531 | 3.34E-05 | **** |
| Cellulosilyticum_lentocellum | 0.00065526 | 0 | 0.01485021 | * |
| Coprococcus_catus | 0 | 0.03973133 | 0.09422328 | ns |
| Coprococcus_comes | 0 | 0.03508451 | 0.05943183 | ns |
| Dorea_formicigenerans | 0.04285237 | 0.08900965 | 0.06739244 | ns |
| Dorea_longicatena | 0 | 0.06663425 | 0.00020297 | *** |
| Dorea_unclassified | 0.03242684 | 0.0641392 | 0.32589978 | ns |
| Lachnoanaerobaculum_saburreum | 5.92E-05 | 0.00019363 | 0.89781595 | ns |
| Lachnospiraceae_bacterium_1_1_57FAA | 0 | 0.00450416 | 0.00167143 | ** |
| Lachnospiraceae_bacterium_1_4_56FAA | 0.00063211 | 0.0117608 | 0.00120412 | ** |
| Lachnospiraceae_bacterium_2_1_58FAA | 0.00028816 | 0.12955965 | 0.05226363 | ns |
| Lachnospiraceae_bacterium_3_1_46FAA | 0 | 0.00606363 | 0.07479878 | ns |
| Lachnospiraceae_bacterium_3_1_57FAA_CT1 | 0 | 0.00124531 | 0.19115076 | ns |
| Lachnospiraceae_bacterium_4_1_37FAA | 5.26E-06 | 0.0005162 | 0.94275983 | ns |
| Lachnospiraceae_bacterium_5_1_57FAA | 0 | 0.00326735 | 0.04723641 | * |
| Lachnospiraceae_bacterium_5_1_63FAA | 0.09151711 | 0.13379469 | 0.98375533 | ns |
| Lachnospiraceae_bacterium_6_1_63FAA | 7.79E-05 | 0.00010142 | 0.42932315 | ns |
| Lachnospiraceae_bacterium_7_1_58FAA | 0.01853737 | 0.01872708 | 0.19270454 | ns |
| Lachnospiraceae_bacterium_8_1_57FAA | 0 | 0.01012168 | 0.00574183 | ** |
| Lachnospiraceae_bacterium_9_1_43BFAA | 0.00117763 | 0.00148319 | 0.31342197 | ns |
| Lachnospiraceae_bacterium_ICM7 | 0.00807447 | 0.0003685 | 0.00066007 | *** |
| Lachnospiraceae_bacterium_oral_taxon_082 | 0.00070316 | 0.0005754 | 0.83403151 | ns |
| Lachnospiraceae_oral_taxon_107 | 8.53E-05 | 7.61E-06 | 0.41678942 | ns |
| Oribacterium_sinus | 0.00192526 | 0.00968177 | 0.0334363 | * |
| Oribacterium_sp_oral_taxon_078 | 1.34E-05 | 0.0001008 | 0.2022382 | ns |
| Roseburia_hominis | 0.0001979 | 0.23405655 | 0.03716841 | * |
| Roseburia_intestinalis | 0.11603816 | 0.64214894 | 0.01784427 | * |
| Roseburia_inulinivorans | 0.19478053 | 1.0664792 | 0.00360212 | ** |
| Roseburia_unclassified | 0 | 0.0663731 | 0.00275721 | ** |
| Shuttleworthia_satelles | 0.01449105 | 0.00046018 | 0.00528086 | ** |
| Stomatobaculum_longum | 0.0002529 | 0.00288416 | 0.03159262 | * |
| Oscillibacter_unclassified | 0 | 0.14030522 | 8.77E-05 | **** |
| Filifactor_alocis | 0 | 9.56E-06 | 0.41678942 | ns |
| Clostridium_bartlettii | 0.21070684 | 0.03160009 | 0.57857807 | ns |
| Clostridium_difficile | 0.06752184 | 0.00539797 | 0.65802018 | ns |
| Clostridium_glycolicum | 0.00011921 | 1.95E-05 | 0.09719861 | ns |
| Clostridium_sordellii | 0 | 2.92E-06 | 0.57231948 | ns |
| Peptostreptococcaceae_noname_unclassified | 0.05517684 | 0.03112664 | 0.05151654 | ns |
| Peptostreptococcus_anaerobius | 0 | 0.00026929 | 0.24452623 | ns |
| Peptostreptococcus_stomatis | 0.00155184 | 0.00196425 | 0.30422338 | ns |
| Peptostreptococcus_unclassified | 0.00091368 | 0.00033912 | 0.21322175 | ns |
| Anaerotruncus_colihominis | 0 | 0.01486257 | 0.05943183 | ns |
| Anaerotruncus_unclassified | 0 | 0.00017655 | 0.31622332 | ns |
| Faecalibacterium_prausnitzii | 0.57969053 | 5.91657257 | 3.71E-10 | **** |
| Ruminococcaceae_bacterium_D16 | 0 | 0.06499558 | 0.09422328 | ns |
| Ruminococcus_bromii | 0 | 0.57646584 | 0.03753781 | * |
| Ruminococcus_callidus | 0 | 0.00298531 | 0.41678942 | ns |
| Ruminococcus_lactaris | 0 | 0.06765239 | 0.31622332 | ns |
| Ruminococcus_sp_5_1_39BFAA | 0 | 0.00821407 | 0.31622332 | ns |
| Subdoligranulum_sp_4_3_54A2FAA | 0 | 0.00757363 | 0.57231948 | ns |
| Subdoligranulum_unclassified | 0.02588447 | 1.55181522 | 1.44E-10 | **** |
| Subdoligranulum_variabile | 0 | 4.53E-05 | 0.31622332 | ns |
| Bulleidia_extructa | 0 | 2.49E-05 | 0.24453154 | ns |
| Catenibacterium_mitsuokai | 0.00354421 | 0 | 0.0874182 | ns |
| Coprobacillus_sp_29_1 | 0.04122842 | 0.04692558 | 0.88506787 | ns |
| Coprobacillus_unclassified | 1.35341947 | 0.47306097 | 0.01211648 | * |
| Eggerthia_catenaformis | 0.00033632 | 0.00035531 | 0.89742011 | ns |
| Clostridium_innocuum | 2.63E-05 | 0.00257292 | 7.32E-05 | **** |
| Clostridium_ramosum | 0.74829316 | 0.11940177 | 0.00928166 | ** |
| Clostridium_spiroforme | 0.03387895 | 0.03200841 | 0.35719466 | ns |
| Erysipelotrichaceae_bacterium_21_3 | 0.01102605 | 0.00781496 | 0.79205994 | ns |
| Erysipelotrichaceae_bacterium_2_2_44A | 0.01016 | 0.00673115 | 0.06321611 | ns |
| Erysipelotrichaceae_bacterium_3_1_53 | 0 | 0.0115931 | 0.11889073 | ns |
| Erysipelotrichaceae_bacterium_6_1_45 | 0.00437895 | 0.04115743 | 0.00118579 | ** |
| Eubacterium_biforme | 0 | 0.00675504 | 0.57231948 | ns |
| Eubacterium_cylindroides | 0 | 0.00040885 | 0.57231948 | ns |
| Eubacterium_dolichum | 0 | 0.00309726 | 0.09422328 | ns |
| Holdemania_filiformis | 0 | 0.00141354 | 0.24453154 | ns |
| Holdemania_sp_AP2 | 0 | 0.01828982 | 0.24453154 | ns |
| Holdemania_unclassified | 0 | 0.0009223 | 0.19115076 | ns |
| Solobacterium_moorei | 0.00939079 | 0.04672982 | 0.0004365 | *** |
| Turicibacter_sanguinis | 0.00052421 | 0.00010097 | 0.73608712 | ns |
| Turicibacter_unclassified | 0.00183579 | 0.0005408 | 0.52507106 | ns |
| Acidaminococcus_fermentans | 0.33698763 | 0.00647301 | 4.52E-08 | **** |
| Acidaminococcus_intestini | 0.02070947 | 0.0134146 | 0.00978396 | ** |
| Acidaminococcus_sp_D21 | 0 | 0.02686991 | 0.19115076 | ns |
| Acidaminococcus_unclassified | 0 | 0.28105761 | 0.0236617 | * |
| Anaeroglobus_geminatus | 0.00364132 | 0.01357637 | 0.04550758 | * |
| Centipeda_periodontii | 0.00018632 | 7.05E-05 | 0.25083607 | ns |
| Dialister_invisus | 0.01119132 | 0.96442345 | 0.03885843 | * |
| Dialister_micraerophilus | 0 | 1.95E-06 | 0.57231948 | ns |
| Dialister_succinatiphilus | 0 | 0.1474377 | 0.24453154 | ns |
| Megamonas_funiformis | 5.79E-06 | 0.71439717 | 0.60786293 | ns |
| Megamonas_hypermegale | 0 | 0.04490159 | 0.11889073 | ns |
| Megamonas_rupellensis | 0 | 0.08536929 | 0.11889073 | ns |
| Megamonas_unclassified | 7.32E-05 | 1.77480832 | 0.07570803 | ns |
| Megasphaera_elsdenii | 0.03760658 | 0.03441212 | 0.33365198 | ns |
| Megasphaera_micronuciformis | 0.25672 | 1.01427248 | 0.00081221 | *** |
| Megasphaera_unclassified | 0.91311421 | 1.40100699 | 0.92758888 | ns |
| Mitsuokella_unclassified | 0.00036947 | 0.00036637 | 0.63726613 | ns |
| Selenomonas_artemidis | 0 | 6.40E-05 | 0.41678942 | ns |
| Selenomonas_flueggei | 0.00357842 | 0.00012646 | 0.39808342 | ns |
| Selenomonas_infelix | 0.00025868 | 1.50E-05 | 0.09367395 | ns |
| Selenomonas_noxia | 0.00109737 | 0.00224177 | 0.8695601 | ns |
| Selenomonas_sputigena | 0.00080184 | 7.76E-05 | 0.41839542 | ns |
| Veillonella_atypica | 0.93458079 | 1.99011903 | 0.00183261 | ** |
| Veillonella_dispar | 0.18247079 | 0.1810046 | 0.329907 | ns |
| Veillonella_parvula | 5.22663237 | 2.36702956 | 0.05102799 | ns |
| Veillonella_sp_6_1_27 | 0 | 0.03048814 | 0.24453154 | ns |
| Veillonella_sp_oral_taxon_158 | 0 | 0.00372443 | 0.57231948 | ns |
| Veillonella_sp_oral_taxon_780 | 0 | 0.00054867 | 0.57231948 | ns |
| Veillonella_unclassified | 6.95530737 | 6.10534363 | 0.59047402 | ns |
| Cetobacterium_somerae | 0 | 0.08905602 | 0.15042816 | ns |
| Fusobacterium_mortiferum | 1.06431053 | 0.46734142 | 0.90897508 | ns |
| Fusobacterium_nucleatum | 0.01399342 | 0.0246285 | 0.10104414 | ns |
| Fusobacterium_periodonticum | 0.00621895 | 0.01920991 | 0.96993004 | ns |
| Fusobacterium_ulcerans | 0.00514079 | 0.00592735 | 0.93087639 | ns |
| Fusobacterium_varium | 0 | 0.00115797 | 0.11889073 | ns |
| Leptotrichia_buccalis | 0 | 2.31E-05 | 0.31622332 | ns |
| Leptotrichia_hofstadii | 0 | 6.11E-06 | 0.31622332 | ns |
| Leptotrichia_shahii | 0.00013132 | 0.00016628 | 0.64376065 | ns |
| Leptotrichia_unclassified | 0.00186053 | 0.00249858 | 0.97427482 | ns |
| Leptotrichia_wadei | 0.00170947 | 0.00214106 | 0.21227167 | ns |
| Brevundimonas_diminuta | 0 | 3.53E-05 | 0.41678942 | ns |
| Brevundimonas_unclassified | 0 | 8.10E-05 | 0.41678942 | ns |
| Ochrobactrum_anthropi | 0 | 5.45E-05 | 0.57231948 | ns |
| Agrobacterium_tumefaciens | 0 | 9.73E-06 | 0.57231948 | ns |
| Agrobacterium_unclassified | 0 | 3.10E-05 | 0.57231948 | ns |
| Shinella_zoogloeoides | 0 | 4.45E-05 | 0.31622332 | ns |
| Sinorhizobium_unclassified | 0 | 2.92E-05 | 0.31622332 | ns |
| Paracoccus_denitrificans | 0 | 0.00046363 | 0.24453154 | ns |
| Paracoccus_unclassified | 0 | 0.0005354 | 0.24453154 | ns |
| Sphingopyxis_unclassified | 1.84E-06 | 3.12E-05 | 0.98155411 | ns |
| Oligella_unclassified | 0 | 4.30E-05 | 0.57231948 | ns |
| Oligella_ureolytica | 0 | 4.35E-05 | 0.41678942 | ns |
| Pusillimonas_unclassified | 0 | 7.52E-06 | 0.57231948 | ns |
| Lautropia_mirabilis | 0.01597368 | 0.0089031 | 0.52313684 | ns |
| Burkholderiales_bacterium_1_1_47 | 0.00738079 | 0.04390681 | 0.01462626 | * |
| Thiomonas_unclassified | 0 | 4.12E-05 | 0.57231948 | ns |
| Alicycliphilus_denitrificans | 0 | 2.26E-05 | 0.31622332 | ns |
| Alicycliphilus_unclassified | 0 | 0.00012867 | 0.57231948 | ns |
| Comamonas_unclassified | 0.00064526 | 0.00476266 | 0.82223493 | ns |
| Delftia_acidovorans | 0 | 0.0001677 | 0.41678942 | ns |
| Delftia_unclassified | 0 | 9.47E-05 | 0.31622332 | ns |
| Limnohabitans_unclassified | 0 | 3.98E-06 | 0.57231948 | ns |
| Polaromonas_unclassified | 1.66E-05 | 0 | 0.0874182 | ns |
| Verminephrobacter_unclassified | 0 | 1.36E-05 | 0.57231948 | ns |
| Parasutterella_excrementihominis | 0.00016263 | 0.03363894 | 0.01207022 | * |
| Sutterella_parvirubra | 0 | 0.13804876 | 0.0236617 | * |
| Sutterella_wadsworthensis | 0.46555342 | 0.20657345 | 0.59383903 | ns |
| Eikenella_corrodens | 0.00788553 | 0.00052115 | 0.26617482 | ns |
| Kingella_denitrificans | 0 | 5.62E-05 | 0.41678942 | ns |
| Kingella_oralis | 0 | 1.95E-06 | 0.57231948 | ns |
| Kingella_unclassified | 2.71E-05 | 0.00008 | 0.62570087 | ns |
| Neisseria_bacilliformis | 0.00014105 | 2.34E-05 | 0.24453154 | ns |
| Neisseria_elongata | 0.00180895 | 0.00064867 | 0.01262296 | * |
| Neisseria_flavescens | 0.00733632 | 0.00918602 | 0.34182607 | ns |
| Neisseria_gonorrhoeae | 0 | 2.39E-06 | 0.57231948 | ns |
| Neisseria_lactamica | 1.55E-05 | 4.07E-06 | 0.41678942 | ns |
| Neisseria_macacae | 0.0001579 | 0.00097797 | 0.20873119 | ns |
| Neisseria_meningitidis | 0.00050079 | 0.00081292 | 0.25066173 | ns |
| Neisseria_sicca | 0.00170079 | 0.00387531 | 0.07765418 | ns |
| Neisseria_sp_oral_taxon_014 | 0 | 0.00227797 | 0.57231948 | ns |
| Neisseria_subflava | 0.00024763 | 0.00026726 | 0.50161122 | ns |
| Neisseria_unclassified | 0.01402895 | 0.03932814 | 0.4304495 | ns |
| Thauera_terpenica | 0 | 0.00059797 | 0.24453154 | ns |
| Desulfobulbus_sp_oral_taxon_041 | 0 | 1.95E-05 | 0.57231948 | ns |
| Bilophila_unclassified | 0.09794579 | 0.20333832 | 0.08478035 | ns |
| Bilophila_wadsworthia | 0.00616421 | 0.01733221 | 0.14156794 | ns |
| Desulfovibrio_desulfuricans | 0 | 0.00179487 | 0.31622332 | ns |
| Arcobacter_unclassified | 0 | 0.00035239 | 0.19115076 | ns |
| Campylobacter_concisus | 0.01816553 | 0.01560522 | 0.68669682 | ns |
| Campylobacter_curvus | 0.00043526 | 0.00076885 | 0.48431235 | ns |
| Campylobacter_gracilis | 0.003915 | 0.01107982 | 0.86197906 | ns |
| Campylobacter_hominis | 0 | 0.00200221 | 0.31622332 | ns |
| Campylobacter_rectus | 0 | 7.96E-06 | 0.57231948 | ns |
| Campylobacter_showae | 0.00372211 | 0.00117274 | 4.83E-05 | **** |
| Campylobacter_ureolyticus | 0 | 1.34E-05 | 0.57231948 | ns |
| Helicobacter_bilis | 0 | 0.00854832 | 0.41678942 | ns |
| Helicobacter_pylori | 0.00078895 | 0 | 0.01485021 | * |
| Aeromonas_caviae | 0 | 7.26E-06 | 0.57231948 | ns |
| Aeromonas_unclassified | 0 | 0.00012584 | 0.31622332 | ns |
| Aeromonas_veronii | 0 | 0.00148752 | 0.41678942 | ns |
| Succinatimonas_hippei | 0 | 4.87E-05 | 0.57231948 | ns |
| Alishewanella_unclassified | 0 | 1.04E-05 | 0.41678942 | ns |
| Cardiobacterium_hominis | 0.00077368 | 0.00038646 | 0.21495325 | ns |
| Cardiobacterium_valvarum | 0 | 7.35E-05 | 0.09422328 | ns |
| Citrobacter_freundii | 0.44406 | 0.02100841 | 0.00076475 | *** |
| Citrobacter_koseri | 0 | 0.00048929 | 0.57231948 | ns |
| Citrobacter_unclassified | 0.34157658 | 0.01331398 | 0.0005343 | *** |
| Citrobacter_youngae | 5.79E-06 | 0 | 0.0874182 | ns |
| Cronobacter_sakazakii | 0 | 0.00011797 | 0.31622332 | ns |
| Cronobacter_unclassified | 0 | 4.69E-06 | 0.41678942 | ns |
| Enterobacteriaceae_bacterium_9_2_54FAA | 0 | 0.00237504 | 0.41678942 | ns |
| Enterobacter_aerogenes | 0.00297316 | 0.01784239 | 0.67974817 | ns |
| Enterobacter_cancerogenus | 0 | 8.87E-05 | 0.57231948 | ns |
| Enterobacter_cloacae | 0.98256316 | 0.01666717 | 0.00144001 | ** |
| Enterobacter_mori | 9.47E-06 | 1.46E-05 | 0.42932315 | ns |
| Enterobacter_sp_MGH_8 | 0 | 0.00095221 | 0.57231948 | ns |
| Escherichia_coli | 14.5830574 | 10.511014 | 0.35547011 | ns |
| Escherichia_fergusonii | 0.00113316 | 0.00013593 | 0.41678942 | ns |
| Escherichia_unclassified | 3.09875053 | 1.81829062 | 0.32846674 | ns |
| Hafnia_alvei | 0 | 0.0010208 | 0.57231948 | ns |
| Klebsiella_oxytoca | 0.12156342 | 0.01178363 | 2.72E-05 | **** |
| Klebsiella_pneumoniae | 11.4054584 | 3.02173035 | 0.00762883 | ** |
| Klebsiella_sp_MS_92_3 | 0.00197421 | 0.00391195 | 0.76298624 | ns |
| Klebsiella_unclassified | 0.19135974 | 0.02897159 | 0.01820899 | * |
| Klebsiella_variicola | 0.00063579 | 0.00011319 | 0.00092252 | *** |
| Morganella_morganii | 0 | 0.00497681 | 0.02981505 | * |
| Pantoea_agglomerans | 0.00046947 | 0 | 0.0874182 | ns |
| Pantoea_unclassified | 0.00115763 | 4.31E-05 | 0.25083607 | ns |
| Proteus_mirabilis | 0.00330553 | 0.00364186 | 0.64376065 | ns |
| Proteus_penneri | 0 | 0.00022947 | 0.31622332 | ns |
| Proteus_unclassified | 0 | 0.00012089 | 0.24453154 | ns |
| Providencia_rettgeri | 0 | 0.00015717 | 0.57231948 | ns |
| Providencia_unclassified | 0 | 9.33E-05 | 0.57231948 | ns |
| Rahnella_aquatilis | 0.00106553 | 0 | 0.01485021 | * |
| Rahnella_unclassified | 0.00694474 | 0 | 0.01485021 | * |
| Raoultella_ornithinolytica | 0.00277368 | 0.03859416 | 0.03731777 | * |
| Salmonella_enterica | 0.05055763 | 0.00224664 | 0.04354406 | * |
| Salmonella_unclassified | 0.00311053 | 0.0002477 | 0.01752116 | * |
| Serratia_liquefaciens | 0 | 3.07E-05 | 0.41678942 | ns |
| Shigella_sonnei | 0 | 0.00071133 | 0.41678942 | ns |
| Yersinia_enterocolitica | 0 | 0.00155283 | 0.57231948 | ns |
| Actinobacillus_unclassified | 0.00275026 | 0.0049915 | 0.39808342 | ns |
| Aggregatibacter_segnis | 0.01882263 | 0.01135876 | 0.80810174 | ns |
| Aggregatibacter_unclassified | 0.0004879 | 0.0035031 | 0.22399215 | ns |
| Haemophilus_haemolyticus | 0.00419842 | 0.00420451 | 0.16392036 | ns |
| Haemophilus_influenzae | 0.00232237 | 0.00025938 | 0.09969352 | ns |
| Haemophilus_parahaemolyticus | 0.00071737 | 0.00561832 | 0.28948744 | ns |
| Haemophilus_parainfluenzae | 0.71298342 | 0.96823336 | 0.55865168 | ns |
| Haemophilus_paraphrohaemolyticus | 0.00091316 | 0.00107646 | 0.07213892 | ns |
| Haemophilus_sputorum | 0.00578211 | 0.00908097 | 0.74648136 | ns |
| Acinetobacter_bereziniae | 2.08E-05 | 0 | 0.0874182 | ns |
| Acinetobacter_guillouiae | 0 | 4.65E-05 | 0.57231948 | ns |
| Acinetobacter_johnsonii | 0 | 0.0003054 | 0.24453154 | ns |
| Acinetobacter_junii | 0 | 8.14E-06 | 0.57231948 | ns |
| Acinetobacter_pittii_calcoaceticus_nosocomialis | 1.47E-05 | 1.20E-05 | 0.42932315 | ns |
| Acinetobacter_soli | 0 | 4.16E-06 | 0.57231948 | ns |
| Acinetobacter_unclassified | 0 | 0.00025646 | 0.24453154 | ns |
| Enhydrobacter_aerosaccus | 0 | 0.00419133 | 0.24453154 | ns |
| Pseudomonas_caeni | 0 | 0.00175319 | 0.19115076 | ns |
| Pseudomonas_fragi | 0.00031158 | 0 | 0.01485021 | * |
| Pseudomonas_unclassified | 0.30785974 | 0.00041186 | 0.04868091 | * |
| Stenotrophomonas_maltophilia | 0 | 3.50E-05 | 0.57231948 | ns |
| Stenotrophomonas_unclassified | 0 | 2.67E-05 | 0.57231948 | ns |
| Treponema_denticola | 0 | 7.35E-06 | 0.57231948 | ns |
| Fretibacterium_fastidiosum | 1.05E-06 | 6.17E-05 | 0.98155411 | ns |
| Pyramidobacter_piscolens | 0 | 0.00960257 | 0.04723641 | * |
| Akkermansia_muciniphila | 0 | 1.13278363 | 0.00275721 | ** |

ns, no significance * p<0.05 ** p<0.01 *** p<0.001

| Table S4 Differential metabolic pathways between HE group or noHE Group | | | | | |
| --- | --- | --- | --- | --- | --- |
| Feature | noHE | HE |  | P-value | Q-value |
| PWY-6113: superpathway of mycolate biosynthesis | 28.05 | 3.664 | 24.386 | 7.66E-10 | 3.72E-07 |
| PWY-5177: glutaryl-CoA degradation | 59.29 | 14.65 | 44.64 | 1.17E-08 | 2.83E-06 |
| TRNA-CHARGING-PWY: tRNA charging | 185 | 90.87 | 94.13 | 1.03E-07 | 1.67E-05 |
| PWY-7209: superpathway of pyrimidine ribonucleosides degradation | 15.1 | 2.796 | 12.304 | 3.74E-07 | 3.03E-05 |
| P562-PWY: myo-inositol degradation I | 0.2298 | 0.6596 | -0.4298 | 1.42E-06 | 9.85E-05 |
| URSIN-PWY: ureide biosynthesis | 0.3363 | 1.73 | -1.3937 | 2.36E-06 | 0.0001434 |
| LPSSYN-PWY: superpathway of lipopolysaccharide biosynthesis | 7.441 | 31.48 | -24.039 | 4.28E-06 | 0.0001889 |
| PWY-5154: L-arginine biosynthesis III (via N-acetyl-L-citrulline) | 145.8 | 230.5 | -84.7 | 4.21E-06 | 0.0001889 |
| PWY-6545: pyrimidine deoxyribonucleotides de novo biosynthesis III | 116 | 58.72 | 57.28 | 3.64E-06 | 0.0001889 |
| PWY-6737: starch degradation V | 384.8 | 213.3 | 171.5 | 6.33E-06 | 0.0002563 |
| PWYG-321: mycolate biosynthesis | 75.37 | 37.72 | 37.65 | 9.53E-06 | 0.0003564 |
| PWY-4361: S-methyl-5-thio-&alpha;-D-ribose 1-phosphate degradation | 0.09948 | 0.966 | -0.86652 | 1.25E-05 | 0.000434 |
| PWY-7270: L-methionine salvage cycle II (plants) | 0 | 0.1999 | -0.1999 | 1.75E-05 | 0.0005659 |
| PWY-6151: S-adenosyl-L-methionine cycle I | 390.5 | 266.4 | 124.1 | 2.65E-05 | 0.0008034 |
| PWY-6630: superpathway of L-tyrosine biosynthesis | 93.62 | 159.8 | -66.18 | 2.91E-05 | 0.0008308 |
| LACTOSECAT-PWY: lactose and galactose degradation I | 30.71 | 62.97 | -32.26 | 3.25E-05 | 0.0008786 |
| COBALSYN-PWY: adenosylcobalamin salvage from cobinamide I | 158.1 | 85.63 | 72.47 | 3.51E-05 | 0.0008971 |
| PWY-5747: 2-methylcitrate cycle II | 5.66 | 16.52 | -10.86 | 3.80E-05 | 0.0009234 |
| PWY-5173: superpathway of acetyl-CoA biosynthesis | 24.8 | 64.79 | -39.99 | 4.14E-05 | 0.0009588 |
| P108-PWY: pyruvate fermentation to propanoate I | 6.946 | 0.08708 | 6.85892 | 4.42E-05 | 0.0009766 |
| ANAGLYCOLYSIS-PWY: glycolysis III (from glucose) | 365.9 | 276.1 | 89.8 | 4.81E-05 | 0.001016 |
| PWY66-389: phytol degradation | 4.351 | 19.19 | -14.839 | 5.04E-05 | 0.00102 |
| PWY-4041: &gamma;-glutamyl cycle | 60.55 | 136.7 | -76.15 | 5.82E-05 | 0.001048 |
| PWY-7013: L-1,2-propanediol degradation | 39.39 | 115.1 | -75.71 | 5.57E-05 | 0.001048 |
| PWY0-42: 2-methylcitrate cycle I | 6.16 | 16.94 | -10.78 | 5.65E-05 | 0.001048 |
| P122-PWY: heterolactic fermentation | 26.8 | 67.22 | -40.42 | 6.48E-05 | 0.001124 |
| PWY-1042: glycolysis IV (plant cytosol) | 409 | 302.4 | 106.6 | 7.57E-05 | 0.001226 |
| PWY-4321: L-glutamate degradation IV | 0.1805 | 1.842 | -1.6615 | 7.38E-05 | 0.001226 |
| PPGPPMET-PWY: ppGpp biosynthesis | 44.75 | 85.93 | -41.18 | 0.0001179 | 0.001848 |
| HSERMETANA-PWY: L-methionine biosynthesis III | 68.13 | 37.42 | 30.71 | 0.0001428 | 0.002168 |
| DTDPRHAMSYN-PWY: dTDP-L-rhamnose biosynthesis I | 272.1 | 198.4 | 73.7 | 0.0001504 | 0.002215 |
| PWY-3801: sucrose degradation II (sucrose synthase) | 0.2323 | 1.872 | -1.6397 | 0.0001647 | 0.002287 |
| PWY-7345: superpathway of anaerobic sucrose degradation | 0.2171 | 1.769 | -1.5519 | 0.0001647 | 0.002287 |
| PWY-5367: petroselinate biosynthesis | 20.25 | 4.047 | 16.203 | 0.0001928 | 0.002603 |
| PWY-7117: C4 photosynthetic carbon assimilation cycle, PEPCK type | 6.311 | 15.65 | -9.339 | 0.0002287 | 0.003004 |
| PWY0-1241: ADP-L-glycero-&beta;-D-manno-heptose biosynthesis | 46.81 | 90.65 | -43.84 | 0.0002463 | 0.003149 |
| TEICHOICACID-PWY: teichoic acid (poly-glycerol) biosynthesis | 0.1846 | 0.193 | -0.0084 | 0.0003058 | 0.003811 |
| PWY-6396: superpathway of 2,3-butanediol biosynthesis | 0.1126 | 1.65 | -1.5374 | 0.0003235 | 0.00393 |
| NONMEVIPP-PWY: methylerythritol phosphate pathway I | 390.1 | 292 | 98.1 | 0.0003431 | 0.004068 |
| GLUCUROCAT-PWY: superpathway of &beta;-D-glucuronide and D-glucuronate degradation | 136.4 | 84.59 | 51.81 | 0.000431 | 0.004988 |
| AST-PWY: L-arginine degradation II (AST pathway) | 25.01 | 55.08 | -30.07 | 0.0004446 | 0.005025 |
| PWY-6837: fatty acid beta-oxidation V (unsaturated, odd number, di-isomerase-dependent) | 0.1511 | 1.125 | -0.9739 | 0.0004761 | 0.005259 |
| PANTOSYN-PWY: pantothenate and coenzyme A biosynthesis I | 246 | 187.9 | 58.1 | 0.0004903 | 0.005295 |
| P161-PWY: acetylene degradation | 52.37 | 93.93 | -41.56 | 0.0007001 | 0.007397 |
| PWY-6901: superpathway of glucose and xylose degradation | 99.65 | 140.6 | -40.95 | 0.0007223 | 0.007469 |
| REDCITCYC: TCA cycle VIII (helicobacter) | 30.31 | 52.66 | -22.35 | 0.0007687 | 0.007783 |
| PWY0-1277: 3-phenylpropanoate and 3-(3-hydroxyphenyl)propanoate degradation | 30.88 | 69.95 | -39.07 | 0.0009882 | 0.009802 |
| PWY-5179: toluene degradation V (aerobic) (via toluene-cis-diol) | 0.01271 | 0.3559 | -0.34319 | 0.001043 | 0.01014 |
| GALACT-GLUCUROCAT-PWY: superpathway of hexuronide and hexuronate degradation | 129.4 | 84.48 | 44.92 | 0.001102 | 0.0105 |
| NAD-BIOSYNTHESIS-II: NAD salvage pathway II | 63.07 | 115.4 | -52.33 | 0.001153 | 0.01078 |
| THISYNARA-PWY: superpathway of thiamin diphosphate biosynthesis III (eukaryotes) | 166.4 | 128.3 | 38.1 | 0.001207 | 0.01106 |
| HCAMHPDEG-PWY: 3-phenylpropanoate and 3-(3-hydroxyphenyl)propanoate degradation to 2-oxopent-4-enoate | 22.88 | 55.85 | -32.97 | 0.001398 | 0.01211 |
| PWY-5083: NAD/NADH phosphorylation and dephosphorylation | 74.16 | 121.4 | -47.24 | 0.001391 | 0.01211 |
| PWY-6690: cinnamate and 3-hydroxycinnamate degradation to 2-oxopent-4-enoate | 22.88 | 55.85 | -32.97 | 0.001398 | 0.01211 |
| UBISYN-PWY: superpathway of ubiquinol-8 biosynthesis (prokaryotic) | 36.43 | 68.41 | -31.98 | 0.00142 | 0.01211 |
| PROTOCATECHUATE-ORTHO-CLEAVAGE-PWY: protocatechuate degradation II (ortho-cleavage pathway) | 4.403 | 22.26 | -17.857 | 0.001601 | 0.0127 |
| PWY-5667: CDP-diacylglycerol biosynthesis I | 426.2 | 339.5 | 86.7 | 0.001648 | 0.0127 |
| PWY-5855: ubiquinol-7 biosynthesis (prokaryotic) | 38.47 | 73.94 | -35.47 | 0.001623 | 0.0127 |
| PWY-5856: ubiquinol-9 biosynthesis (prokaryotic) | 38.47 | 73.94 | -35.47 | 0.001623 | 0.0127 |
| PWY-5857: ubiquinol-10 biosynthesis (prokaryotic) | 38.47 | 73.94 | -35.47 | 0.001623 | 0.0127 |
| PWY-6708: ubiquinol-8 biosynthesis (prokaryotic) | 38.47 | 73.94 | -35.47 | 0.001623 | 0.0127 |
| PWY-6803: phosphatidylcholine acyl editing | 49.98 | 89.79 | -39.81 | 0.001672 | 0.0127 |
| PWY0-1319: CDP-diacylglycerol biosynthesis II | 426.5 | 340.8 | 85.7 | 0.001722 | 0.01288 |
| PWY-7528: L-methionine salvage cycle I (bacteria and plants) | 0.04646 | 0.3351 | -0.28864 | 0.001795 | 0.01322 |
| PWY-7357: thiamin formation from pyrithiamine and oxythiamine (yeast) | 254.8 | 195.7 | 59.1 | 0.001879 | 0.01343 |
| PWY0-1297: superpathway of purine deoxyribonucleosides degradation | 133.2 | 176.1 | -42.9 | 0.001879 | 0.01343 |
| SULFATE-CYS-PWY: superpathway of sulfate assimilation and cysteine biosynthesis | 75.41 | 116.3 | -40.89 | 0.002006 | 0.01413 |
| ALLANTOINDEG-PWY: superpathway of allantoin degradation in yeast | 0.8265 | 1.977 | -1.1505 | 0.0022 | 0.01517 |
| METHGLYUT-PWY: superpathway of methylglyoxal degradation | 22.13 | 37.63 | -15.5 | 0.002216 | 0.01517 |
| PWY-5913: TCA cycle VI (obligate autotrophs) | 38.04 | 67.99 | -29.95 | 0.002266 | 0.0153 |
| ASPASN-PWY: superpathway of L-aspartate and L-asparagine biosynthesis | 255.5 | 200.7 | 54.8 | 0.002365 | 0.01575 |
| GLYCOLYSIS-E-D: superpathway of glycolysis and Entner-Doudoroff | 80.43 | 115.1 | -34.67 | 0.002539 | 0.01668 |
| P23-PWY: reductive TCA cycle I | 3.906 | 12.16 | -8.254 | 0.002641 | 0.01689 |
| PWY3DJ-35471: L-ascorbate biosynthesis IV | 0 | 0.3949 | -0.3949 | 0.002641 | 0.01689 |
| PWY-5918: superpathay of heme biosynthesis from glutamate | 44.74 | 75.56 | -30.82 | 0.002822 | 0.01758 |
| PWY-6519: 8-amino-7-oxononanoate biosynthesis I | 63.49 | 97.28 | -33.79 | 0.002802 | 0.01758 |
| PWY-5675: nitrate reduction V (assimilatory) | 1.357 | 3.704 | -2.347 | 0.002944 | 0.01811 |
| FUCCAT-PWY: fucose degradation | 47.73 | 85.55 | -37.82 | 0.003112 | 0.01867 |
| HEME-BIOSYNTHESIS-II: heme biosynthesis I (aerobic) | 37.6 | 62.3 | -24.7 | 0.00309 | 0.01867 |
| GLYOXYLATE-BYPASS: glyoxylate cycle | 55.24 | 91.79 | -36.55 | 0.003267 | 0.01872 |
| ILEUSYN-PWY: L-isoleucine biosynthesis I (from threonine) | 546.7 | 432.5 | 114.2 | 0.003312 | 0.01872 |
| PWY-5181: toluene degradation III (aerobic) (via p-cresol) | 0.08033 | 0.1482 | -0.06787 | 0.003226 | 0.01872 |
| PWY-724: superpathway of L-lysine, L-threonine and L-methionine biosynthesis II | 323.6 | 254.5 | 69.1 | 0.003312 | 0.01872 |
| VALSYN-PWY: L-valine biosynthesis | 546.7 | 432.5 | 114.2 | 0.003312 | 0.01872 |
| BIOTIN-BIOSYNTHESIS-PWY: biotin biosynthesis I | 69.65 | 102.9 | -33.25 | 0.003452 | 0.01928 |
| P125-PWY: superpathway of (R,R)-butanediol biosynthesis | 1.719 | 2.523 | -0.804 | 0.003509 | 0.01938 |
| FASYN-INITIAL-PWY: superpathway of fatty acid biosynthesis initiation (E. coli) | 77.91 | 111.6 | -33.69 | 0.003598 | 0.01943 |
| PWY-5028: L-histidine degradation II | 0.01062 | 0.2462 | -0.23558 | 0.003581 | 0.01943 |
| PWY-6628: superpathway of L-phenylalanine biosynthesis | 141.2 | 194.6 | -53.4 | 0.003698 | 0.01953 |
| PWY-7388: octanoyl-[acyl-carrier protein] biosynthesis (mitochondria, yeast) | 72.32 | 107.4 | -35.08 | 0.003698 | 0.01953 |
| PWY0-862: (5Z)-dodec-5-enoate biosynthesis | 81.82 | 119.6 | -37.78 | 0.003749 | 0.01959 |
| PWY-5415: catechol degradation I (meta-cleavage pathway) | 6.354 | 30.1 | -23.746 | 0.004223 | 0.02183 |
| PWY-7664: oleate biosynthesis IV (anaerobic) | 83.14 | 118.9 | -35.76 | 0.004353 | 0.02227 |
| FASYN-ELONG-PWY: fatty acid elongation -- saturated | 85.65 | 120.5 | -34.85 | 0.004412 | 0.02233 |
| CALVIN-PWY: Calvin-Benson-Bassham cycle | 328.4 | 261.7 | 66.7 | 0.004532 | 0.02247 |
| PWY-5103: L-isoleucine biosynthesis III | 333.7 | 237.4 | 96.3 | 0.004532 | 0.02247 |
| PWY-7196: superpathway of pyrimidine ribonucleosides salvage | 63.06 | 31.42 | 31.64 | 0.00476 | 0.02337 |
| PWY-7111: pyruvate fermentation to isobutanol (engineered) | 555.5 | 454.6 | 100.9 | 0.005318 | 0.02559 |
| PWY0-1261: anhydromuropeptides recycling | 132 | 181.9 | -49.9 | 0.005318 | 0.02559 |
| HEXITOLDEGSUPER-PWY: superpathway of hexitol degradation (bacteria) | 97.72 | 125 | -27.28 | 0.005606 | 0.02645 |
| SO4ASSIM-PWY: sulfate reduction I (assimilatory) | 81.35 | 121.4 | -40.05 | 0.005569 | 0.02645 |
| PWY-6282: palmitoleate biosynthesis I (from (5Z)-dodec-5-enoate) | 77.42 | 109.7 | -32.28 | 0.005681 | 0.02655 |
| PWY-5686: UMP biosynthesis | 520.6 | 424.2 | 96.4 | 0.005832 | 0.02699 |
| FERMENTATION-PWY: mixed acid fermentation | 49.15 | 76.03 | -26.88 | 0.006065 | 0.02781 |
| KETOGLUCONMET-PWY: ketogluconate metabolism | 25.74 | 62.17 | -36.43 | 0.006143 | 0.0279 |
| PWY-5723: Rubisco shunt | 73.84 | 105.1 | -31.26 | 0.006226 | 0.02802 |
| PWY-7269: NAD/NADP-NADH/NADPH mitochondrial interconversion (yeast) | 53.82 | 82.59 | -28.77 | 0.006468 | 0.02884 |
| P221-PWY: octane oxidation | 0.06391 | 0.2445 | -0.18059 | 0.006928 | 0.03061 |
| PWY-5973: cis-vaccenate biosynthesis | 231.3 | 183.8 | 47.5 | 0.007365 | 0.03196 |
| PWY-7527: L-methionine salvage cycle III | 0.1498 | 1.164 | -1.0142 | 0.007318 | 0.03196 |
| PWY-6471: peptidoglycan biosynthesis IV (Enterococcus faecium) | 7.15 | 15.26 | -8.11 | 0.007714 | 0.03318 |
| GLYCOLYSIS-TCA-GLYOX-BYPASS: superpathway of glycolysis, pyruvate dehydrogenase, TCA, and glyoxylate bypass | 71.47 | 108.2 | -36.73 | 0.0078 | 0.03325 |
| PWY-5862: superpathway of demethylmenaquinol-9 biosynthesis | 59.16 | 86.37 | -27.21 | 0.007985 | 0.03375 |
| PWY-5860: superpathway of demethylmenaquinol-6 biosynthesis I | 59.2 | 86.37 | -27.17 | 0.008143 | 0.03411 |
| PWY-6892: thiazole biosynthesis I (E. coli) | 66.31 | 91.73 | -25.42 | 0.008363 | 0.03474 |
| GLUCOSE1PMETAB-PWY: glucose and glucose-1-phosphate degradation | 62.37 | 93.31 | -30.94 | 0.009133 | 0.03689 |
| PWY-5845: superpathway of menaquinol-9 biosynthesis | 72.39 | 101.8 | -29.41 | 0.00906 | 0.03689 |
| PWY-5850: superpathway of menaquinol-6 biosynthesis I | 72.44 | 101.8 | -29.36 | 0.009235 | 0.03689 |
| PWY-5896: superpathway of menaquinol-10 biosynthesis | 72.39 | 101.8 | -29.41 | 0.00906 | 0.03689 |
| PWY-7094: fatty acid salvage | 0.02576 | 0.1688 | -0.14304 | 0.009261 | 0.03689 |
| PWY-7242: D-fructuronate degradation | 145.2 | 99.84 | 45.36 | 0.009365 | 0.037 |
| PWY-6284: superpathway of unsaturated fatty acids biosynthesis (E. coli) | 19.82 | 9.252 | 10.568 | 0.009635 | 0.03776 |
| PWY0-1415: superpathway of heme biosynthesis from uroporphyrinogen-III | 29.98 | 44.87 | -14.89 | 0.01093 | 0.0425 |
| PWY-1861: formaldehyde assimilation II (RuMP Cycle) | 0.2388 | 0.6818 | -0.443 | 0.0112 | 0.04297 |
| PWY-821: superpathway of sulfur amino acid biosynthesis (Saccharomyces cerevisiae) | 11.99 | 9.752 | 2.238 | 0.01132 | 0.04297 |
| THREOCAT-PWY: superpathway of L-threonine metabolism | 0.1963 | 2.303 | -2.1067 | 0.01128 | 0.04297 |
| BRANCHED-CHAIN-AA-SYN-PWY: superpathway of branched amino acid biosynthesis | 354.6 | 264.1 | 90.5 | 0.01198 | 0.04514 |
| GLUCARDEG-PWY: D-glucarate degradation I | 34.14 | 61.21 | -27.07 | 0.01257 | 0.04687 |
| PWY-5138: unsaturated, even numbered fatty acid &beta;-oxidation | 9.348 | 22.74 | -13.392 | 0.01273 | 0.04687 |
| PWY-6121: 5-aminoimidazole ribonucleotide biosynthesis I | 420.8 | 344.1 | 76.7 | 0.01273 | 0.04687 |
| GALACTUROCAT-PWY: D-galacturonate degradation I | 122.4 | 84.83 | 37.57 | 0.01288 | 0.04701 |
| P105-PWY: TCA cycle IV (2-oxoglutarate decarboxylase) | 63.49 | 97.37 | -33.88 | 0.01296 | 0.04701 |
| COA-PWY: coenzyme A biosynthesis I | 247.3 | 205 | 42.3 | 0.01401 | 0.04797 |
| GLUCONEO-PWY: gluconeogenesis I | 49.37 | 83.03 | -33.66 | 0.01352 | 0.04797 |
| PANTO-PWY: phosphopantothenate biosynthesis I | 343 | 292 | 51 | 0.01435 | 0.04797 |
| POLYAMINSYN3-PWY: superpathway of polyamine biosynthesis II | 5.381 | 2.074 | 3.307 | 0.01385 | 0.04797 |
| PWY-5081: L-tryptophan degradation VIII (to tryptophol) | 0 | 0.00746 | -0.00746 | 0.01441 | 0.04797 |
| PWY-5101: L-isoleucine biosynthesis II | 25.89 | 26.73 | -0.84 | 0.01408 | 0.04797 |
| PWY-6891: thiazole biosynthesis II (Bacillus) | 51.77 | 70.08 | -18.31 | 0.01336 | 0.04797 |
| PWY-7165: L-ascorbate biosynthesis VI (engineered pathway) | 0 | 0.2139 | -0.2139 | 0.01441 | 0.04797 |
| PWY-7288: fatty acid &beta;-oxidation (peroxisome, yeast) | 0.01203 | 0.05385 | -0.04182 | 0.01419 | 0.04797 |
| PWY-7391: isoprene biosynthesis II (engineered) | 0.02317 | 0.106 | -0.08283 | 0.01376 | 0.04797 |
| PWY66-391: fatty acid &beta;-oxidation VI (peroxisome) | 0.02179 | 0.0866 | -0.06481 | 0.01419 | 0.04797 |
| TCA-GLYOX-BYPASS: superpathway of glyoxylate bypass and TCA | 59.56 | 95.78 | -36.22 | 0.01418 | 0.04797 |
| NAGLIPASYN-PWY: lipid IVA biosynthesis | 67.56 | 92.71 | -25.15 | 0.01452 | 0.04801 |
| PWY-5861: superpathway of demethylmenaquinol-8 biosynthesis | 65.2 | 90.93 | -25.73 | 0.01478 | 0.0482 |
| PWY-6969: TCA cycle V (2-oxoglutarate:ferredoxin oxidoreductase) | 87.15 | 124.2 | -37.05 | 0.0147 | 0.0482 |
| DENOVOPURINE2-PWY: superpathway of purine nucleotides de novo biosynthesis II | 157.9 | 120.1 | 37.8 | 0.01523 | 0.04934 |
| PWY-7219: adenosine ribonucleotides de novo biosynthesis | 699.4 | 580.2 | 119.2 | 0.01615 | 0.05198 |
| PWY-5692: allantoin degradation to glyoxylate II | 0.2635 | 0.07417 | 0.18933 | 0.01642 | 0.05216 |
| URDEGR-PWY: superpathway of allantoin degradation in plants | 0.2635 | 0.07417 | 0.18933 | 0.01642 | 0.05216 |
| PWY-6467: Kdo transfer to lipid IVA III (Chlamydia) | 0.8836 | 4.017 | -3.1334 | 0.01658 | 0.05232 |
| POLYISOPRENSYN-PWY: polyisoprenoid biosynthesis (E. coli) | 34.16 | 50.27 | -16.11 | 0.01712 | 0.05329 |
| PWY-5838: superpathway of menaquinol-8 biosynthesis I | 78.92 | 106.3 | -27.38 | 0.01722 | 0.05329 |
| PWY3O-355: stearate biosynthesis III (fungi) | 0.00501 | 0.09033 | -0.08532 | 0.01722 | 0.05329 |
| TCA: TCA cycle I (prokaryotic) | 94.33 | 138.3 | -43.97 | 0.01732 | 0.05329 |
| P461-PWY: hexitol fermentation to lactate, formate, ethanol and acetate | 91.33 | 120.7 | -29.37 | 0.01753 | 0.05357 |
| P164-PWY: purine nucleobases degradation I (anaerobic) | 13.58 | 10.41 | 3.17 | 0.0184 | 0.05401 |
| PWY-5505: L-glutamate and L-glutamine biosynthesis | 13.87 | 11.62 | 2.25 | 0.0178 | 0.05401 |
| PWY-5514: UDP-N-acetyl-D-galactosamine biosynthesis II | 0.007602 | 0.03456 | -0.026958 | 0.01844 | 0.05401 |
| PWY-5656: mannosylglycerate biosynthesis I | 7.931 | 2.553 | 5.378 | 0.01874 | 0.05401 |
| PWY-6122: 5-aminoimidazole ribonucleotide biosynthesis II | 429.2 | 348.4 | 80.8 | 0.01879 | 0.05401 |
| PWY-6138: CMP-N-acetylneuraminate biosynthesis I (eukaryotes) | 0.002184 | 0.04817 | -0.045986 | 0.01914 | 0.05401 |
| PWY-6277: superpathway of 5-aminoimidazole ribonucleotide biosynthesis | 429.2 | 348.4 | 80.8 | 0.01879 | 0.05401 |
| PWY-6318: L-phenylalanine degradation IV (mammalian, via side chain) | 0.5603 | 0.5367 | 0.0236 | 0.01896 | 0.05401 |
| PWY-7411: superpathway of phosphatidate biosynthesis (yeast) | 0.01053 | 0.03742 | -0.02689 | 0.01908 | 0.05401 |
| PWY0-1061: superpathway of L-alanine biosynthesis | 106.8 | 144.1 | -37.3 | 0.01922 | 0.05401 |
| PWY0-881: superpathway of fatty acid biosynthesis I (E. coli) | 0.03175 | 0.1125 | -0.08075 | 0.01844 | 0.05401 |
| PWY66-375: leukotriene biosynthesis | 0.01294 | 0.01881 | -0.00587 | 0.01844 | 0.05401 |
| PWY66-422: D-galactose degradation V (Leloir pathway) | 208 | 155.6 | 52.4 | 0.01857 | 0.05401 |
| UDPNACETYLGALSYN-PWY: UDP-N-acetyl-D-glucosamine biosynthesis II | 0.01096 | 0.03294 | -0.02198 | 0.01908 | 0.05401 |
| P441-PWY: superpathway of N-acetylneuraminate degradation | 89.51 | 114.1 | -24.59 | 0.01967 | 0.05463 |
| PWY-5676: acetyl-CoA fermentation to butanoate II | 53.07 | 73.81 | -20.74 | 0.01966 | 0.05463 |
| PWY-7039: phosphatidate metabolism, as a signaling molecule | 6.672 | 17.34 | -10.668 | 0.01978 | 0.05463 |
| PWY-7187: pyrimidine deoxyribonucleotides de novo biosynthesis II | 141.4 | 109.7 | 31.7 | 0.02013 | 0.05526 |
| FUC-RHAMCAT-PWY: superpathway of fucose and rhamnose degradation | 58.15 | 83.07 | -24.92 | 0.02024 | 0.05526 |
| PWY-7385: 1,3-propanediol biosynthesis (engineered) | 0.05129 | 0.1964 | -0.14511 | 0.02237 | 0.06075 |
| GALACTARDEG-PWY: D-galactarate degradation I | 27.55 | 51.96 | -24.41 | 0.02533 | 0.06802 |
| GLUCARGALACTSUPER-PWY: superpathway of D-glucarate and D-galactarate degradation | 27.55 | 51.96 | -24.41 | 0.02533 | 0.06802 |
| PWY0-1296: purine ribonucleosides degradation | 327.3 | 278 | 49.3 | 0.02549 | 0.06806 |
| PWY0-781: aspartate superpathway | 91.82 | 117.1 | -25.28 | 0.02606 | 0.0692 |
| PWY-7294: xylose degradation IV | 0.0259 | 1.172 | -1.1461 | 0.02638 | 0.06967 |
| CATECHOL-ORTHO-CLEAVAGE-PWY: catechol degradation to &beta;-ketoadipate | 2.144 | 2.316 | -0.172 | 0.02893 | 0.07599 |
| PWY66-367: ketogenesis | 0.5091 | 0.03861 | 0.47049 | 0.03166 | 0.08272 |
| PWY-5840: superpathway of menaquinol-7 biosynthesis | 80.98 | 103.5 | -22.52 | 0.03326 | 0.08597 |
| PWY-6317: galactose degradation I (Leloir pathway) | 197.9 | 141.9 | 56 | 0.03308 | 0.08597 |
| PWY-6897: thiamin salvage II | 210.7 | 182.9 | 27.8 | 0.03344 | 0.08599 |
| PWY-6263: superpathway of menaquinol-8 biosynthesis II | 4.648 | 0.9052 | 3.7428 | 0.03505 | 0.08965 |
| PWY-5659: GDP-mannose biosynthesis | 142.1 | 104.2 | 37.9 | 0.03564 | 0.09068 |
| PWY-6470: peptidoglycan biosynthesis V (&beta;-lactam resistance) | 0.03684 | 0.123 | -0.08616 | 0.0363 | 0.09164 |
| PWY-7254: TCA cycle VII (acetate-producers) | 42.03 | 67.86 | -25.83 | 0.03639 | 0.09164 |
| PWY-7211: superpathway of pyrimidine deoxyribonucleotides de novo biosynthesis | 84.99 | 64.44 | 20.55 | 0.03678 | 0.09214 |
| PWY-6182: superpathway of salicylate degradation | 0.0582 | 0.08807 | -0.02987 | 0.03709 | 0.09245 |
| PWY-7220: adenosine deoxyribonucleotides de novo biosynthesis II | 211.8 | 247.3 | -35.5 | 0.03876 | 0.09562 |
| PWY-7222: guanosine deoxyribonucleotides de novo biosynthesis II | 211.8 | 247.3 | -35.5 | 0.03876 | 0.09562 |
| PWY-5920: superpathway of heme biosynthesis from glycine | 11.8 | 29.62 | -17.82 | 0.03964 | 0.09729 |
| PWY-5863: superpathway of phylloquinol biosynthesis | 57.43 | 76.45 | -19.02 | 0.04083 | 0.0997 |
| PWY-7323: superpathway of GDP-mannose-derived O-antigen building blocks biosynthesis | 70.38 | 51.08 | 19.3 | 0.04254 | 0.1034 |
| PWY-6168: flavin biosynthesis III (fungi) | 224.9 | 196.7 | 28.2 | 0.04298 | 0.1039 |
| PWY-6859: all-trans-farnesol biosynthesis | 28.38 | 37 | -8.62 | 0.04342 | 0.1045 |
| NONOXIPENT-PWY: pentose phosphate pathway (non-oxidative branch) | 322.2 | 262.1 | 60.1 | 0.04501 | 0.1078 |
| PWY-5097: L-lysine biosynthesis VI | 430.4 | 369.6 | 60.8 | 0.0457 | 0.1089 |
| PWY-5088: L-glutamate degradation VIII (to propanoate) | 0.2571 | 0 | 0.2571 | 0.04671 | 0.1102 |
| PWY-7279: aerobic respiration II (cytochrome c) (yeast) | 15.17 | 21.05 | -5.88 | 0.04663 | 0.1102 |
| PWY-6386: UDP-N-acetylmuramoyl-pentapeptide biosynthesis II (lysine-containing) | 501.4 | 434.8 | 66.6 | 0.04856 | 0.114 |
| PWY0-1298: superpathway of pyrimidine deoxyribonucleosides degradation | 62.8 | 85.12 | -22.32 | 0.0493 | 0.1152 |
